# Huoshan Dendrobium Zengye Jiedu Formula mitigates radiation-induced oral mucositis and improves oral immune microenvironment by targeting the EGFR/PI3K/AKT pathway: evidence from network pharmacology, molecular docking, and experimental validation

**DOI:** 10.3389/fimmu.2025.1559400

**Published:** 2025-03-10

**Authors:** Chang Liu, Xinru Liu, Jiabao Liu, Hao Zhang, Pengcheng Zhang, Xingxing Huo, Hang Song, Yongfu Zhu

**Affiliations:** ^1^ The First Clinical Medical College, Anhui University of Chinese Medicine, Hefei, China; ^2^ Department of Oncology, The First Affiliated Hospital of Zhejiang Chinese Medical University, Hangzhou, China; ^3^ Experimental Center of Clinical Research, Scientific Research Department, The First Affiliated Hospital of Anhui University of Chinese Medicine, Hefei, China; ^4^ College of Integrated Chinese and Western Medicine, Anhui University of Chinese Medicine, Hefei, China; ^5^ Department of Oncology, The First Affiliated Hospital of Anhui University of Chinese Medicine, Hefei, China; ^6^ Guo-jun Hu Inheritance Talent Training Office, The First Affiliated Hospital of Anhui University of Chinese Medicine, Hefei, China

**Keywords:** radiation-induced oral mucositis, pharmacological mechanism, network pharmacology, molecular docking, experimental validation

## Abstract

**Introduction:**

Radiation-induced oral mucositis (RIOM) manifests as mucosal ulceration, pain, and dysphagia, disrupting treatment and quality of life. Its pathogenesis involves inflammatory imbalance and immune dysregulation, driven by microbial infiltration and cytokine storms. Current therapies remain inadequate, necessitating deeper exploration of immune-microbial interactions for effective interventions.

**Methods:**

Bioactive components of Huoshan Dendrobium Zengye Jiedu Formula (HDZJF) and RIOM-related targets were retrieved from public databases. Core therapeutic targets and pathways were systematically analyzed via protein-protein interaction (PPI) networks, Gene Ontology (GO), and Kyoto Encyclopedia of Genes and Genomes (KEGG) enrichment. Molecular docking evaluated interactions between HDZJF components and key targets. A rat RIOM model validated HDZJF efficacy by assessing mucositis severity, inflammatory cytokines, and EGFR/PI3K/AKT pathway protein expression.

**Results:**

A total of 102 bioactive components and 379 potential targets for RIOM were identified. GO and KEGG enrichment analyses suggest that HDZJF exerts therapeutic effects on RIOM by modulating processes such as angiogenesis, inflammation, and apoptosis through pathways like PI3K-AKT. Molecular docking confirmed strong binding affinities between HDZJF components and key targets. *In vivo*, HDZJF reduced inflammation, promoted mucosal healing, improved body weight, and modulated protein expression related to EGFR/PI3K/AKT.

**Discussion:**

The findings highlight HDZJF's capacity to alleviate RIOM by targeting the EGFR/PI3K/AKT pathway, thereby suppressing inflammatory responses and apoptotic processes. These results underscore HDZJF's translational potential for RIOM treatment and justify further clinical investigation into its therapeutic utility.

## Graphical Abstract

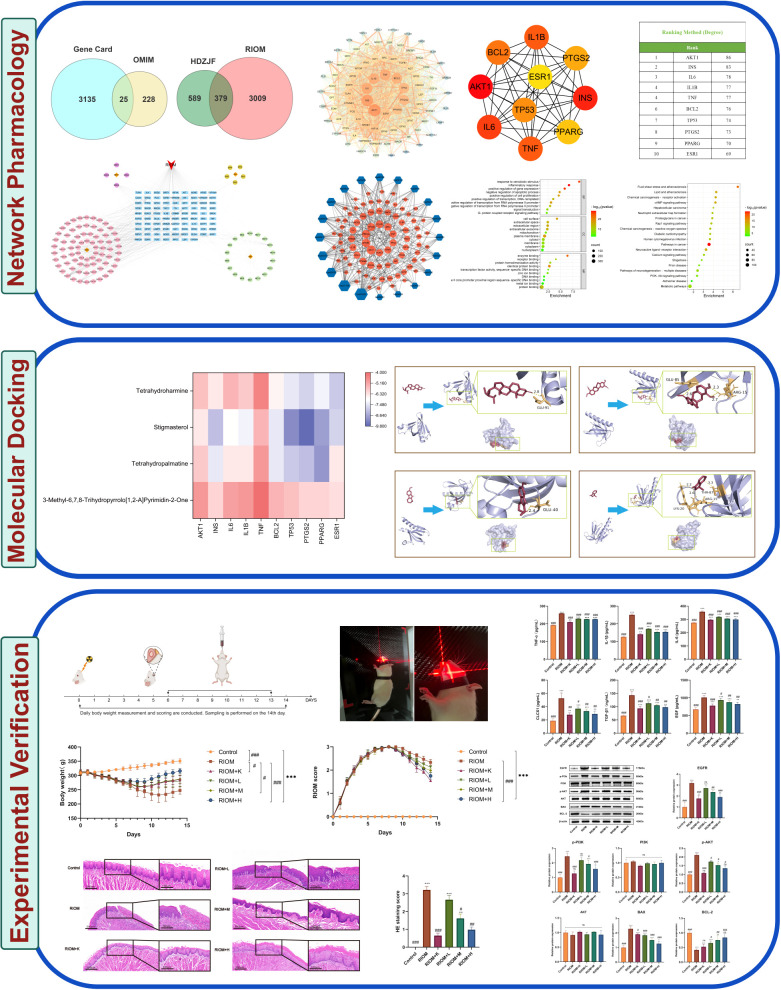

## 1 Introduction

Head and neck tumors contribute to 5% ~ 10% of cancer diagnoses, and radiation therapy plays a crucial role in their treatment. However, it inevitably damages surrounding healthy tissues during treatment (1). Radiation-induced oral mucositis (RIOM) is a common adverse consequence following radiotherapy for head and neck malignancies. Clinically, it is characterized by oral mucosal erythema, edema, erosion, and ulceration. Patients often experience varying degrees of pain and difficulty swallowing. In severe cases, eating and drinking are significantly affected, and some patients are forced to discontinue treatment due to unbearable pain (2, 3).According to reports from the World Health Organization (WHO), the incidence of grade III or IV oral mucositis among patients receiving head and neck radiotherapy (e.g., 60-70 Gy) approaches 85%. However, nearly all treated patients exhibit varying levels of RIOM (4). The onset of RIOM has a profound effect on both the patient’s overall health and the effectiveness of treatment (5).

It has been demonstrated that the emergence and healing of RIOM are intricately linked to inflammatory processes and immune responses (6). During the exceedingly painful ulcerative phase, oral bacteria infiltrate the ulcers, prompting neighboring cells to release a cascade of cytokines and chemokines. These signaling molecules attract inflammatory cells, including macrophages, mast cells, and neutrophils, which subsequently produce an additional surge of proinflammatory molecules. This domino effect further exacerbates cellular apoptosis and tissue injury (7). However, a concurrent healing process is initiated through the interaction between the extracellular matrix, submucosal mesenchymal cells, and innate immune cells. This collaborative effort stimulates epithelial cell proliferation and differentiation, while also facilitating the restoration of a balanced oral microbial community (8, 9). Thus, the complex interplay between innate immune defenses and the oral microbiome emerges as a crucial factor in understanding the underlying mechanisms of RIOM.

Various treatment options are currently implemented to address RIOM, including standardized oral hygiene, antimicrobial agents, anti-inflammatory treatments, growth factors, analgesic drugs, and oral rinses (10). Medications such as corticosteroids, cimetidine, and amitriptyline are commonly used to alleviate inflammation and pain associated with RIOM. However, continued administration of these substances could lead to several negative outcomes, such as fungal infections, rashes, sleepiness, tingling or burning feelings, and exhaustion (11). Therefore, effective measures are needed to reduce the incidence and severity of RIOM in order to alleviate patient suffering.

Traditional Chinese medicine (TCM), a core component of Chinese medicine with over 2,000 years of history, plays an indispensable role in disease prevention and treatment. Recently, more patients with RIOM have sought out TCM, drawn to its substantial therapeutic effects and relatively few adverse reactions (12). Studies have shown that several herbs and natural compounds with therapeutic effects—such as reducing inflammation, combating oxidative stress, and alleviating pain—hold promise for treating RIOM (13–17). Kangfuxin Liquid is an ethanol extract derived from the American cockroach, known for its effects in promoting blood circulation, nourishing yin, and facilitating muscle regeneration. Research has shown that Kangfuxin Liquid can help prevent RIOM and alleviate the severity of oral pain to some extent. As a result, it is often used as a control medication in both animal experiments and clinical observations (18, 19).

Huoshan Dendrobium Zengye Jiedu Formula (HDZJF) is a contemporary adaptation of the traditional Zengye Decoction. It includes four medicinal herbs: Dendrobium huoshanense (Chinese name: Huoshan Shihu), Ophiopogon japonicus (Chinese name: Maidong), Rehmannia glutinosa (Chinese name: Shengdihuang), and Glycyrrhiza uralensis (Chinese name: Shenggancao). This modern formulation substitutes Scrophularia ningpoensis (Chinese name: Xuanshen) with Dendrobium huoshanense (Chinese name: Huoshan Shihu) and Glycyrrhiza uralensis (Chinese name: Shenggancao) (20). The formulation has the ability to nourish yin, clear heat, and tonify deficiency, providing targeted support for individuals with malignancies. Numerous studies have shown that the method of nourishing yin and clearing heat has a beneficial therapeutic effect on RIOM (21, 22). Meanwhile, this modified formula has shown promising results in our institution for treating oral mucositis caused by chemotherapy and radiotherapy. Clinical observations suggest that HDZJF alleviates local inflammation in RIOM patients and improves conditions like redness, tissue damage, ulcers, and discomfort of the oral mucosa. It is currently under patent application, with the application number 2024102698967.

However, the underlying mechanism of action of HDZJF in treating RIOM remains incompletely understood. Considering the wide variety of active substances in TCM, a synergistic effect through multiple mechanisms is likely. It is essential to identify appropriate methods for analyzing these components and their respective targets.

Network pharmacology, an integrated research approach combining cheminformatics, bioinformatics, network biology, and traditional pharmacology, uncovers the connections between numerous components and targets in a holistic framework (23). Molecular docking, a virtual screening technique simulating the interaction of small molecule ligands with receptor protein binding sites, has gained increasing popularity in drug development (24).

Numerous studies have employed the Wistar rat RIOM model for animal experiments, owing to the strain’s robust immune response and its susceptibility to radiation-induced tissue damage, making it an ideal subject for investigating radiation-induced mucosal injury. Pathophysiological changes induced in this model, including mucosal inflammation, epithelial cell damage, and cytokine dysregulation, closely resemble the clinical manifestations of oral mucositis observed in head and neck cancer patients undergoing radiotherapy (25, 26). Thus, this animal model is well-suited for the current study.

## 2 Materials and methods

### 2.1 Preparation of the HDZJF aqueous extract

The four medicinal ingredients of HDZJF—Dendrobium huoshanense (Chinese name: Huoshan Shihu), Ophiopogon japonicus (Chinese name: Maidong), Rehmannia glutinosa (Chinese name: Shengdihuang), and Glycyrrhiza uralensis (Chinese name: Shenggancao)—were sourced from the pharmacy of Anhui Provincial Hospital of Traditional Chinese Medicine. The ratio of the ingredients was 10:6:6:5. Ophiopogon japonicus (Chinese name: Maidong), Rehmannia glutinosa (Chinese name: Shengdihuang), and Glycyrrhiza uralensis (Chinese name: Shenggancao) were first soaked in ultrapure water, ensuring that the liquid covered the herbs, and allowed to soak for 30–40 minutes. The mixture was then placed into a decoction pot, with water added to a height of 2–3 cm above the herbs (10 times the weight of the herbs). The herbs were decocted for 30 minutes, with stirring 2–3 times during the process. The resulting filtrate was collected, and the herb residue was decocted a second time, for a shorter period. The filtrates were combined, concentrated to 100 mL, and freeze-dried to obtain a powder. The Huoshan Dendrobium (Chinese name: Huoshan Shihu) was directly ground into powder and passed through a 6-mesh sieve. The freeze-dried powder was then mixed with the powder of Dendrobium huoshanense (Chinese name: Huoshan Shihu) to obtain the HDZJF aqueous extract. The chemical components of HDZJF were identified using Ultra-Performance Liquid Chromatography (UPLC) coupled with High-Resolution Mass Spectrometry (HRMS). Detailed procedures and the characteristic chromatograms are provided in **Supplementary Material S1**.

### 2.2 Identification of key bioactive components and associated targets in HDZJF

The active components of the traditional Chinese medicine formula were retrieved from the BATMAN-TCM database (http://bionet.ncpsb.org.cn/batman-tcm/). The search for the effective ingredients of the HDZJF was conducted using keywords such as Shihu, Shengdihuang, Maidong, and Shenggancao. For the BATMAN-TCM database, filtering criteria of a score ≥ 20 and a *P*-value < 0.05 were applied (27).

### 2.3 Gathering of molecular targets associated with RIOM

Molecular targets associated with RIOM were selected from three sources: GeneCards (https://www.genecards.org), OMIM (https://omim.org), and TTD (https://idrblab.org/ttd/) (28–30). The keyword “Radiation induced oral mucositis” was used to search these databases, and redundant genes were excluded.

### 2.4 Construction of component-target-disease network

The targets associated with RIOM were intersected with those related to the drug, and a Venn diagram was generated to identify the potential targets of HDZJF in combating RIOM. Overlapping target genes between HDZJF-related and RIOM-related targets were imported into the STRING database (http://string-db.org), with “Homo sapiens” selected as the organism and a minimum interaction score of 0.4 to retrieve protein-protein interaction (PPI) data (31). The PPI network was then constructed using Cytoscape 3.10.0 software (32).

### 2.5 Immune infiltration and immune checkpoint analysis

The datasets GSE62395 (33) and GSE62397 (34) for RIOM were obtained from the NCBI database, and samples of RIOM were selected. The oral microenvironment of RIOM was evaluated using 28 immune cell types from the ssGSEA package (35). The GSVA package was used to assess the scores of key intersection genes in the samples, and high and low expression groups were defined based on median values.

### 2.6 GO and KEGG enrichment analysis

KEGG pathway analysis was performed using the DAVID database (https://david.ncifcrf.gov/) (36). GO enrichment analysis was conducted for biological processes (BP), cellular components (CC), and molecular functions (MF). A threshold of *P* < 0.05 and FDR < 0.05 was applied to determine the significance of GO and KEGG enrichment. The results of the GO and KEGG pathway analyses were visualized using the bioinformatics online platform (https://www.bioinformatics.com.cn/) (37). Furthermore, a pathway-target network diagram for KEGG pathway analysis was generated using Cytoscape 3.10.0.

### 2.7 Computational validation of active ingredient-target interactions via molecular docking

Core ingredients and key targets were selected based on the degree values from network analysis. Molecular docking experiments were conducted using software tools such as AutoDock Tools and PyMOL. The 2D structures of small molecules were obtained from the PubChem database (http://zine.docking.org/), and subsequently converted into mol2 files using Chem3D for further use (38). The 3D structural files of key targets were retrieved from the PDB database (https://www.rcsb.org/), downloaded, and prepared in PyMOL, which included basic steps such as extracting small molecules and removing hydrogens (39). The molecules and target proteins were then imported into AutoDock Tools for molecular docking. Finally, PyMOL was used for visualization and analysis of the docking results.

### 2.8 *In vivo* animal experiment

#### 2.8.1 Rats

We obtained 36 male Wistar rats, each with a weight of 300 ± 20 g, from Shanghai Slac Laboratory Animal Co., Ltd. (Shanghai, China). They were kept in the First Affiliated Experimental Animal Research Center of Anhui University of Chinese Medicine (Hefei, China) in a pathogen-free facility. The animals were housed at a temperature of 23 ± 2°C and humidity of 50 ± 10%. They were exposed to 12 hours of light followed by 12 hours of darkness each day, with free access to food and water at all times. All protocols related to animal use were reviewed and authorized in compliance with institutional and national ethical guidelines. All experiments followed the Laboratory Animal Regulations approved by the State Council of China.

#### 2.8.2 Development of the RIOM model and animal grouping

A total of 36 rats, after one week of acclimatization, were randomly assigned to six groups (n = 6 per group) using the envelope method: a Control group, a RIOM model group (RIOM), a Kangfuxin Liquid treatment group (RIOM+K), and three groups receiving low, medium, or high doses of HDZJF (RIOM+L, RIOM+M, RIOM+H). The sample size of six rats per group is a reasonable choice derived from combining experience, statistical power analysis, and ethical and economic considerations. This sample size is widely accepted in many animal experiments and has been proven to be effective.

All rats, except those in the Control group, were anesthetized via intraperitoneal injection of carbamoyl ester at a concentration of 0.2g/ml, with a dosage of 0.006 mL/g. Once fully anesthetized, rats were secured in a lateral position using black adhesive tape and subjected to irradiation in an X-ray biological irradiator (SHARP 100, Raycision Medical Technology Co., Ltd). Irradiation was applied to the left cheek using a cross-axis coordinate system, with the X-axis aligned parallel to the lower nostrils and the Y-axis parallel to the lower jaw. The irradiation diameter was 1.5 cm, the source-to-skin distance was 37 cm, and the dose rate was 2.5 Gy/min at a voltage of 225 kV and a current of 17 mA, with a depth of penetration of 2 cm. This procedure induced RIOM in the tongue tip, middle tongue, and left cheek mucosa. The modeling method and its associated parameters were determined through multiple preliminary trials, enabling the RIOM model rats to not only display typical symptoms but also maintain a high survival rate. The manifestations of the RIOM rats are as follows: Redness in the rats’ tongues was observed from days 1 to 3 post-irradiation, becoming more pronounced by days 4 to 5. By days 5–6, rats in all treatment groups exhibited typical RIOM symptoms.

On day 6 post-irradiation, rats in the RIOM+L, RIOM+M, and RIOM+H groups were administered 2 mL of aqueous extract containing 0.71g/kg, 1.42g/kg, and 2.84g/kg, respectively, by oral gavage once daily for 8 consecutive days. The RIOM+K group received 2 mL of Kangfuxin Liquid, while the remaining groups were treated with 0.9% saline solution at the same volume. Body weight was regularly measured at 9:00 AM every morning throughout the experiment. At the conclusion of the study on day 14, serum and oral mucosal tissues were harvested for further analysis.

During the experiment, only the personnel responsible for drug administration were aware of the group allocations. All other individuals involved in the evaluation and data analysis were blinded to the group assignments of the rats.

#### 2.8.3 Evaluation of RIOM

The RIOM score was assessed by three calibrated and blinded evaluators. All evaluators were trained researchers. Following the methodology described by Lima et al., the macroscopic features such as erythema, hemorrhage, epithelial ulceration, and abscess were evaluated using a 0-3 scoring system (40, 41). The scoring criteria were as follows:

0 points: The mucosal appearance was generally normal, with only slight erythema and mild congestion, if at all. There were no visible signs of hemorrhagic areas, ulcers, or abscesses, indicating an absence of significant pathology.
1 point: Moderate erythema and congestion were evident, suggesting some degree of inflammation. However, there were no detectable hemorrhagic areas, ulcers, or abscesses, which implies a relatively mild condition without severe damage to the mucosal tissue.
2 points: There was marked erythema and congestion, indicating a more pronounced inflammatory response. The presence of hemorrhagic areas, along with small ulcers or scar tissue, suggests a moderate degree of mucosal damage, though no abscess formation was observed.
3 points: Severe erythema and congestion were observed, with significant mucosal damage. Hemorrhagic areas, widespread ulcers, and abscesses were present, reflecting a severe inflammatory process and extensive tissue involvement.

#### 2.8.4 Histopathological assessment

Histopathological analysis of the oral mucosa specimens was performed using hematoxylin and eosin (HE) staining to observe any changes in the tissue. Initially, the samples were preserved by immersion in a 10% formalin solution, ensuring the maintenance of their cellular architecture. After the fixation process, the specimens underwent a series of dehydration steps, gradually transitioning through different concentrations of ethanol, and were then treated with xylene to eliminate any residual water content. Once fully dehydrated, the specimens were embedded in paraffin to preserve their structure for subsequent examination. Thin tissue sections, approximately 5 µm in thickness, were then prepared from the paraffin blocks. These sections were stained with HE and analyzed using a NanoZoomer S60 digital slide scanner (Hamamatsu, Japan) for detailed observation of the tissue’s histological features.

The HE staining scores were also determined via histopathological assessments carried out by three pathology-trained researchers, with the evaluation process conducted in a blinded manner. Histological scores were assigned based on a referenced oral mucosal histopathological grading system, with a 0-4 scale for mucosal damage (42). The scoring criteria were as follows:

0 points: No radiation injury; normal mucosa with no significant changes.
1 point: Focal or diffuse alteration of the basal cell layer with nuclear atypia and ≤ 2 dyskeratotic squamous cells. This indicates mild changes to the mucosal architecture, with limited cellular abnormalities.
2 points: Epithelial thinning (2–4 cell layers) and/or ≥ 3 dyskeratotic squamous cells in the epithelium. This suggests moderate damage to the epithelial layer, with noticeable thinning and a higher number of abnormal cells.
3 points: Loss of epithelium without a break in keratinization or the presence of subepithelial vesicle/bullous formation. This indicates severe tissue damage, either through loss of the epithelial layer with maintained keratinization or the formation of fluid-filled structures beneath the epithelium, reflecting deeper tissue involvement.
4 points: Complete loss of epithelial and keratinized cell layers; ulceration. This represents the most severe injury, with both the epithelial and keratinized layers lost, leading to ulceration and significant tissue destruction.

#### 2.8.5 Enzyme linked immunosorbent assay

Serum samples were carefully collected from rats to assess the levels of several key cytokines, including TNF-α, IL-1β, IL-6, CLCX1, TGF-β, and EGF. To quantify the cytokine concentrations, commercially available enzyme-linked immunosorbent assay (ELISA) kits (Reddy Biosciences, Wuhan) were used, following the manufacturer’s detailed instructions to ensure accurate and reliable measurements.

#### 2.8.6 Western blotting

Tissue proteins were extracted with RIPA buffer (Beyotime, Shanghai, China), separated by SDS-PAGE, and transferred to PVDF membranes (Merck Millipore, MA, USA). After blocking with 5% BSA for 1 hour, the membranes were incubated overnight with primary antibody at 4°C, followed by 1-hour incubation with secondary antibody. Detection was performed using enhanced chemiluminescence. Western blot images were analyzed using ImageJ software (NIH, Bethesda, MD, USA). This study used the following primary antibodies: anti-EGFR (YT1497, IMMUNOWAY), anti-p-PI3K (YM0765, IMMUNOWAY), anti-PI3K (YM8045, IMMUNOWAY), anti-p-AKT (YP0590, IMMUNOWAY), anti-AKT (YM3618, IMMUNOWAY), anti-BAX (YT0455, IMMUNOWAY), anti-BCL-2(YM3041, IMMUNOWAY), and anti-β-actin (66009-1-Ig, SanYing Biotech Co., Ltd.).

### 2.9 Statistical analysis

The data analysis was performed using IBM SPSS Statistics for Windows version 26.0 (IBM Corp., Armonk, USA). Data are presented as mean ± standard deviation (M ± SD). Initially, normality and homogeneity of variance tests were conducted for the multiple groups. If the data met the assumptions of normality and homogeneity of variance, one-way analysis of variance (ANOVA) was applied. Otherwise, the Kruskal-Wallis H test was used. For repeated measures data, normality, homogeneity of variance, and sphericity tests were performed, followed by repeated measures ANOVA. When significant differences were detected, *post-hoc* analyses were conducted using either Fisher’s Least Significant Difference (LSD) or Games-Howell test. Statistical significance was set at p < 0.05 (including p<0.01 and p<0.001).

## 3 Results

### 3.1 Network pharmacology-based analysis

#### 3.1.1 Identification of active components in HDZJF and screening of related targets

HDZJF consists of four traditional Chinese herbs. After eliminating duplicates, 102 active components were identified in total. These active components and their corresponding targets were imported into Cytoscape 3.10.0 software to construct the HDZJF active component-target network, resulting in 968 targets. The ten most effective active components of HDZJF were chosen based on their degree values, which include: 3-Methyl-6,7,8-Trihydropyrrolo[1,2-A]Pyrimidin-2-One, Tetrahydropalmatine, Gamma-Aminobutyric Acid, Stigmasterol, Methylglyoxal, Tetrahydroharmine, Alpha-Trihydroxy Coprostanic Acid, 18alpha-Glycyrrhetinic Acid, Glycyrrhetinic Acid, and Beta-Glycyrrhetinic Acid.

**Figure 1:**
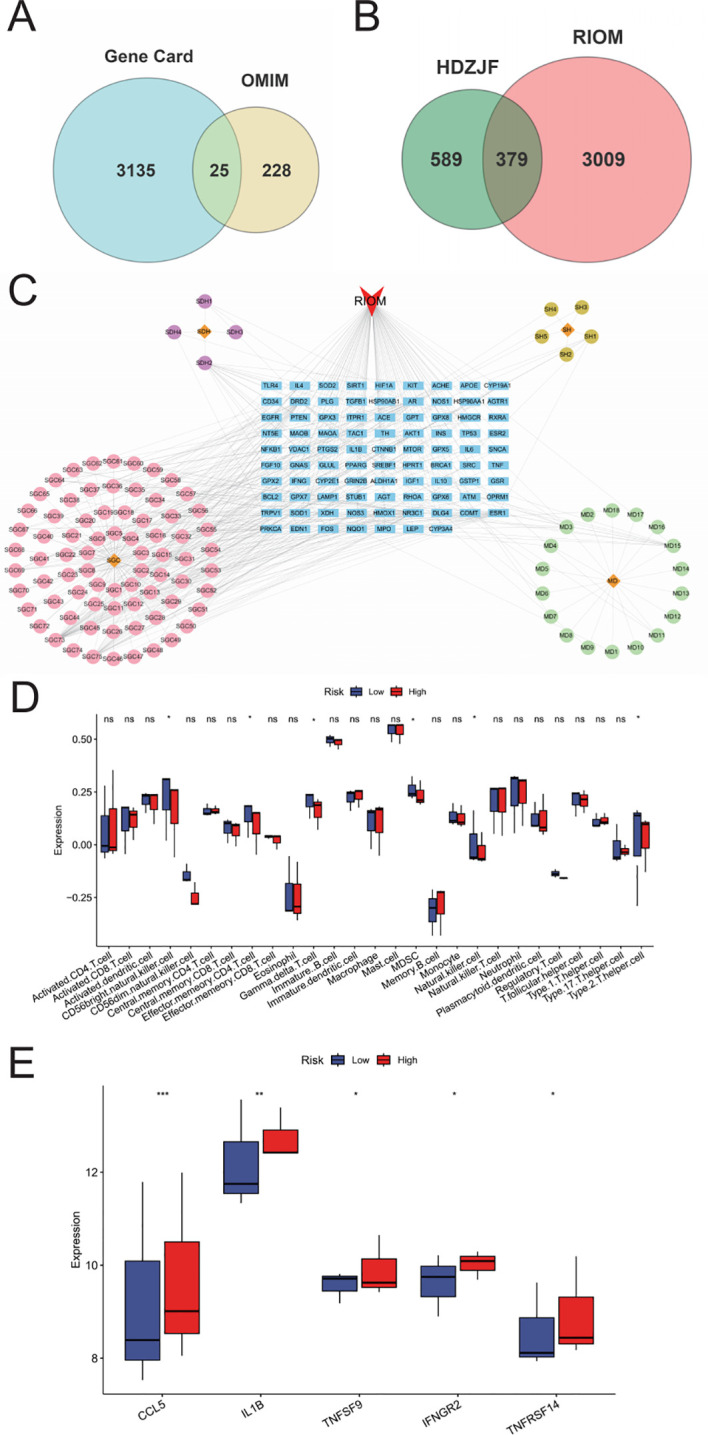
Results of Network Analysis. **(A)** Venn diagram showing the intersection of target points retrieved from GeneCards and OMIM disease databases. **(B)** Venn diagram of active components and disease targets. **(C)** Component-target-disease network of RIOM and HDZJF. **(D)** Bar chart of differences in ssGSEA immune infiltration analysis. **(E)** Bar chart of differences in ssGSEA inflammatory factor expression analysis. *P < 0.05, **P < 0.01, ***P < 0.001, ns: not significant.

As shown in **Figure 1A**, 3388 disease-related targets for RIOM were obtained by searching the GeneCards, OMIM, and TTD databases. Among these, 3160 disease targets were found in GeneCards, 253 in OMIM, and no relevant targets were found in TTD, with 25 duplicate targets identified. The intersection of HDZJF-related targets and RIOM disease targets was obtained, and a Venn diagram was drawn, resulting in 379 overlapping targets. These are considered potential therapeutic targets of HDZJF for RIOM treatment, as shown in **Figure 1B**.

#### 3.1.2 Component-target-disease network and analysis

To explore the potential relationships between the four medicinal plants and their bioactive compounds in HDZJF, as well as the targets associated with RIOM, we constructed a component-target-disease network using Cytoscape 3.10.0 software. The network consists of 194 nodes and 377 edges, as shown in **Figure 1C**. This indicates that HDZJF exerts its effects on RIOM through multiple components and targets.

#### 3.1.3 Analysis of the correlation between oral Immune cells and the inhibition of inflammatory factor expression

We performed immune infiltration analysis using the ssGSEA package, and the results are shown in **Figure 1D**. The high expression group had relatively lower levels of CD56+NK cells, Effector memory CD4 T cells, gd-δ T cells, MDSCs, and Th2 cells, indicating that traditional Chinese medicine may affect functional immune cells in the oral cavity by targeting specific genes to promote disease healing.

Meanwhile, we obtained immune response-related genes, as shown in **Figure 1E**. The high expression group showed high expression of inflammatory factors such as CCL5, IL1B, and TNFSF9, which are involved in cellular inflammatory responses. This suggests that the anti-inflammatory effect of traditional Chinese medicine may inhibit disease development by suppressing the expression of inflammatory factors.

#### 3.1.4 Construction of the PPI network and core target screening

**Figure 2:**
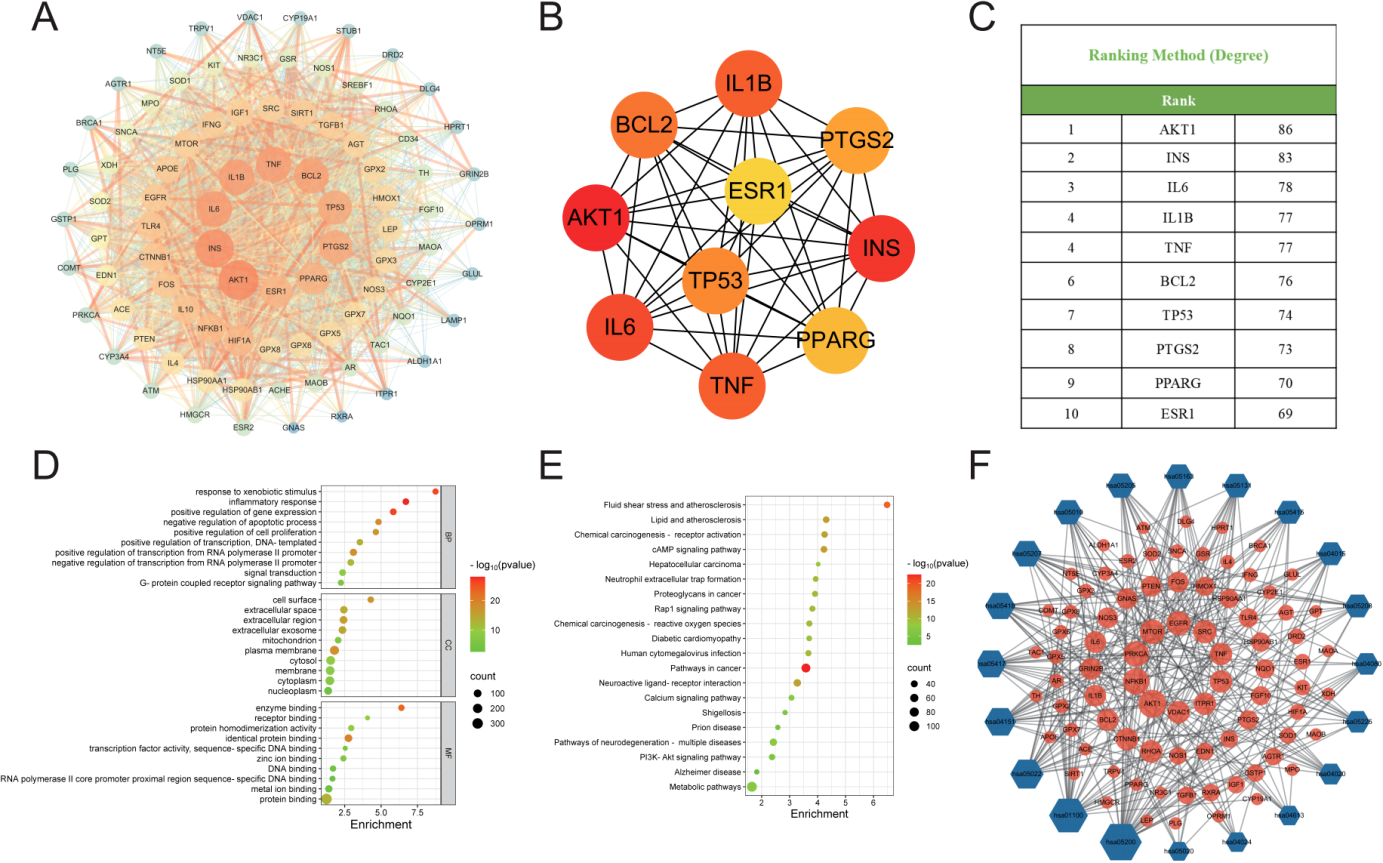
Results of Network Analysis, GO and KEGG Enrichment Analysis. **(A)** Protein-Protein Interaction network, where targets are represented by nodes and protein interactions are represented by edges. The size of the nodes is proportional to their degree. As the color shifts from yellow to red, the node degree increases. **(B)** The top 10 core genes identified from **(A)**. **(C)** Core target list of HDZJF for treating RIOM based on degree centrality. **(D)** The top 10 significantly enriched terms in BP, CC, and MF are shown in panel. **(E)** The top 20 enriched KEGG pathways are presented in panel. **(F)** A target-pathway network illustrating the effects of HDZJF on RIOM is depicted in panel, with blue nodes representing pathways and red nodes representing target genes. The node size is proportional to the degree value.

The intersection targets were imported into the STRING database, and the targets were filtered using Cytoscape 3.10.0 to construct the PPI network of potential therapeutic targets for HDZJF in treating RIOM, as shown in **Figure 2A**. The network consists of 88 nodes and 1,991 edges. By ranking nodes based on their degree values, the core targets (top ten by degree) of HDZJF in RIOM treatment were identified as AKT1, INS, IL-6, IL-1β, TNF, BCL2, TP53, PTGS2, PPARG, and ESR1, as shown in **Figures 2B, C**.

#### 3.1.5 GO and KEGG pathway enrichment analysis and construction of target-pathway network

To verify the biological roles of the potential targets of HDZJF for RIOM, Gene Ontology (GO) enrichment analysis was performed. A total of 839 terms were obtained, including 661 Biological Processes (BP), 80 Cellular Components (CC), and 98 Molecular Functions (MF). The top 10 ranked BP, CC, and MF terms based on the p-value are shown in **Figure 2D**. The top five enriched BP terms were: inflammatory response, positive regulation of gene expression, response to xenobiotic stimulus, negative regulation of apoptotic process, and positive regulation of transcription from RNA polymerase II promoter. The top five enriched CC terms were: plasma membrane, cell surface, extracellular region, extracellular exosome, and extracellular space. The top five enriched MF terms were: enzyme binding, identical protein binding, protein binding, receptor binding, and protein homodimerization activity.

To further investigate the potential mechanisms of HDZJF in relation to RIOM, KEGG pathway enrichment analysis was performed. A total of 189 signaling pathways were identified. The 20 most significant KEGG pathways closely related to RIOM are shown in **Figure 2E**, including: Metabolic pathways, Pathways in cancer, Neuroactive ligand-receptor interaction, Pathways of neurodegeneration - multiple diseases, cAMP signaling pathway, Lipid and atherosclerosis, Fluid shear stress and atherosclerosis, Chemical carcinogenesis - receptor activation, PI3K-Akt signaling pathway, Chemical carcinogenesis - reactive oxygen species, Human cytomegalovirus infection, Proteoglycans in cancer, Rap1 signaling pathway, Calcium signaling pathway, Neutrophil extracellular trap formation, Diabetic cardiomyopathy, Shigellosis, Prion disease, Alzheimer disease, Hepatocellular carcinoma. Based on these top 20 KEGG pathways and their associated genes, a target-pathway network was constructed to elucidate the molecular mechanisms of HDZJF in RIOM, as shown in **Figure 2F**. The pathways with the highest number of associated genes were: hsa05200: Pathways in cancer (33 genes), hsa01100: Metabolic pathways (29 genes), hsa05022: Pathways of neurodegeneration - multiple diseases (22 genes), hsa04151: PI3K-Akt signaling pathway (20 genes), and hsa05417: Lipid and atherosclerosis (19 genes). Among the target genes in these top 20 pathways, AKT1, NFKB1, PRKCA, MTOR, EGFR, SRC, TNF, TP53, ITPR1, and VDAC1 were recognized as key enriched genes. These results suggest that the impact of HDZJF on RIOM could be associated with various pathways.

### 3.2 Binding affinity of active compounds to key targets through molecular docking

**Figure 3:**
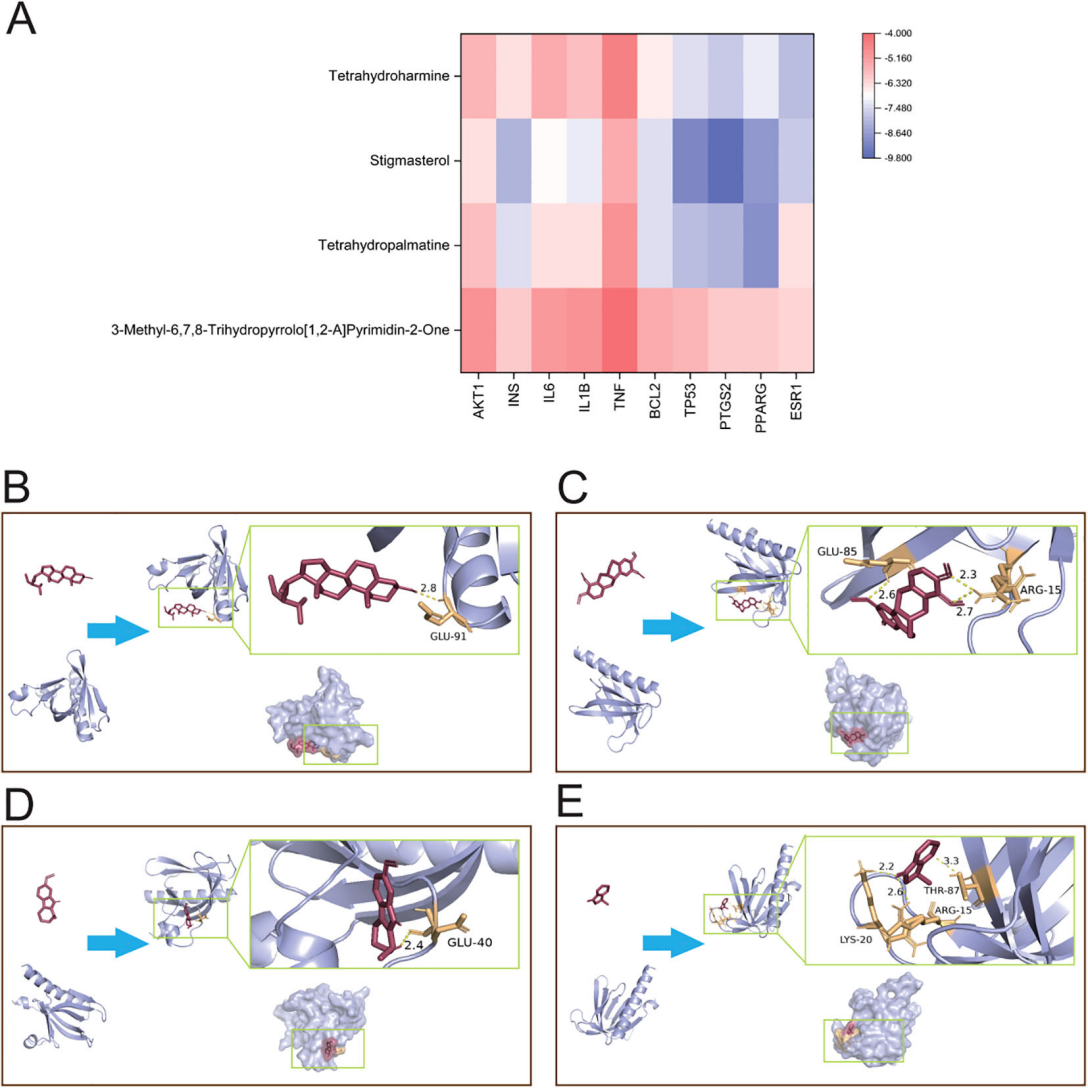
Molecular docking. **(A)** The binding energy heat map of core components and core targets of HDZJF. **(B)** Interaction analysis of AKT1 docking with Stigmasterol. **(C)** Interaction analysis of AKT1 docking with Tetrahydropalmatine. **(D)** Interaction analysis of AKT1 docking with Tetrahydroharmine. **(E)** Interaction analysis of AKT1 docking with 3-Methyl-6,7,8-Trihydropyrrolo[1,2-A]Pyrimidin-2-One.

The binding affinities between 10 core proteins and 4 core ligands were calculated using AutoDock software, as shown in **Figure 3A**. Notably, the binding energy of Stigmasterol with the PTGS2 target was the lowest, at -9.8 kcal/mol. Moreover, the interaction energies of other components with their corresponding targets were generally below -5 kcal/mol. These results suggest that the core components of HDZJF, particularly Stigmasterol, Tetrahydropalmatine, Tetrahydroharmine, and 3-Methyl-6,7,8-Trihydropyrrolo[1,2-A]Pyrimidin-2-One, exhibit high affinity with the core targets.

Finally, based on the network pharmacology results of HDZJF’s effects on the core targets of RIOM, a visualization analysis was performed for AKT1 and the four core ligands: Stigmasterol, Tetrahydropalmatine, Tetrahydroharmine, and 3-Methyl-6,7,8-Trihydropyrrolo[1,2-A]Pyrimidin-2-One. The docking diagrams are presented in **Figures 3B–E**.

### 3.3 *In vivo* animal experiment validation

**Figure 4:**
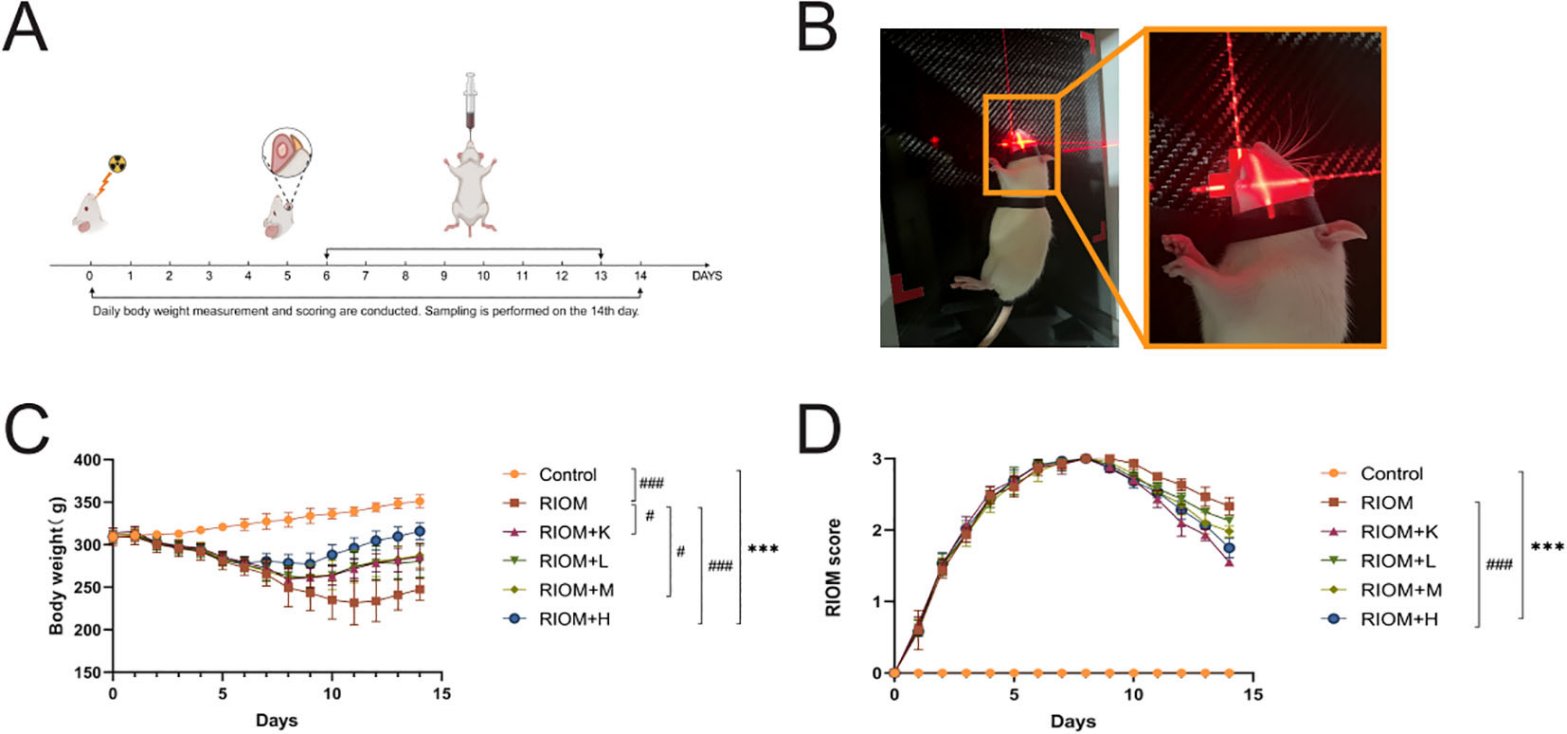
Experimental process and changes in rat body weight and mucositis score. **(A)** Schematic of the Study Design, showing the treatment protocol, modeling process, sampling time points, and data measurements required during the experiment. **(B)** Establishment of the RIOM rat model using an X-ray biological irradiator. **(C)** Body weight, a general health indicator of rats. **(D)** Mucosal healing scores, reflecting the severity of RIOM symptoms in rats. The data are presented as mean ± SEM, with n = 6 per group. *P < 0.05, **P < 0.01, ***P < 0.001 vs. Control; #*P* < 0.05, ##*P* < 0.01, ###*P* < 0.001 vs. RIOM.

To evaluate the therapeutic effects of HDZJF on RIOM in a rat model, an RIOM model was established using an X-ray biological irradiator. The experimental procedure is detailed in **Figures 4A, B**. The study duration was 14 days, with model induction occurring on day 0. HDZJF, Kangfuxin solution, or physiological saline were administered via gavage. Body weight and mucosal healing scores were recorded daily at a consistent time. Tissue collection was performed on day 14. No mortality was observed during the study, and all 36 rats survived until the final tissue collection, when tissue samples were harvested for subsequent analysis.

#### 3.3.1 HDZJF alleviates RIOM symptoms

Body weight is a key indicator of health status. The body weight of rats exposed to radiation was significantly lower than that of the control group, as shown in **Figure 4C**; **Table 1** (*P* < 0.001). However, the weight loss induced by RIOM was significantly alleviated in the RIOM+K, RIOM+M, and RIOM+H groups (*P* < 0.01 or *P* < 0.001).

**Table 1:** Body weight for each group of rats.

Group	Weight(g) (mean ± SEM)	95%CI	F value (repeated measures ANOVA)	p-value
Control	327.83 ± 14.72###	(320.76,334.89)	35.005	p < 0.001
RIOM	267.15 ± 31.59***	(260.08,274.22)		
RIOM+K	284.53 ± 19.38***#	(277.47,291.60)		
RIOM+L	282.65 ± 19.21***	(275.59,289.72)		
RIOM+M	284.33 ± 20.02***#	(277.27,291.40)		
RIOM+H	295.26 ± 15.51***###	(288.19,302.32)		

*P < 0.05, **P < 0.01, ***P < 0.001 vs. Control; #P < 0.05, ##P < 0.01, ###P < 0.001 vs. RIOM.

During the experimental procedure, the oral mucosa of the rats was observed daily, and scores were recorded. The results indicated that, in the RIOM group, the oral mucosa exhibited ulcerative lesions covered by a dense pseudomembrane, with noticeable abscess formation around the affected area. Notably, with the exception of the control group, all rats subjected to X-ray irradiation developed severe RIOM, with the scores reaching the maximum value of 3 on day 8. Treatment with various doses of HDZJF significantly accelerated mucosal healing. As shown in **Figure 4D**; **Table 2**, compared to the RIOM group, the mucosal inflammation scores in the RIOM+L, RIOM+M, RIOM+H, and RIOM+K treatment groups were significantly improved (*P* < 0.001), indicating that HDZJF effectively alleviates the symptoms of RIOM.

**Table 2:** Oral mucositis score for each group on days 9–14.

Group	RIOM score (mean ± SEM)	95%CI	F value (repeated measures ANOVA)	p-value
Control	0.00 ± 0.00###	(-0.051,0.051)	1680.37	p < 0.001
RIOM	2.69 ± 0.25***	(2.64,2.74)		
RIOM+K	2.27 ± 0.47***###	(2.22,2.32)		
RIOM+L	2.51 ± 0.28***###	(2.46,2.56)		
RIOM+M	2.45 ± 0.36***###	(2.40,2.50)		
RIOM+H	2.39 ± 0.39***###	(2.34,2.44)		

*p < 0.05, **p < 0.01, ***p < 0.001 vs. Control; #p < 0.05, ##p < 0.01, ###p < 0.001 vs. RIOM.

#### 3.3.2 HDZJF improves histopathological findings in RIOM

**Figure 5:**
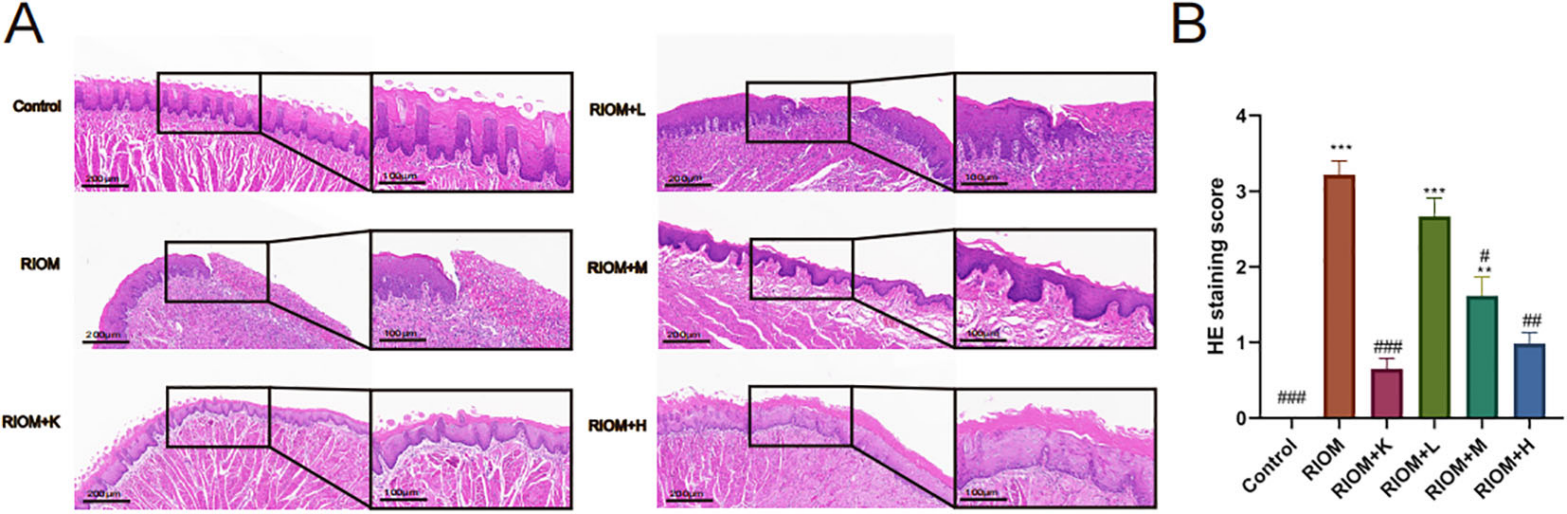
Histopathological analysis. **(A)** HE staining images of rat tongue mucosal tissue (scale bar: 200 and 100 μm). **(B)** HE staining scores for each group. The data are presented as mean ± SEM, with n = 6 per group. *P < 0.05, **P < 0.01, ***P < 0.001 vs. Control; #*P* < 0.05, ##*P* < 0.01, ###*P* < 0.001 vs. RIOM.

Histopathological analysis revealed that the Control group exhibited intact epithelial cells with no inflammatory cell infiltration. In contrast, the RIOM group showed abscess formation, mucosal loss, severe epithelial destruction, and significant inflammatory cell infiltration, along with numerous necrotic cell fragments. The RIOM+K group demonstrated relatively well-organized epithelial cell arrangement with minimal inflammatory cell infiltration, approaching normal mucosal structure. In the RIOM+L group, focal necrosis of the mucosal epithelium was observed, along with structural loss, nuclear condensation, fragmentation, or dissolution. A small amount of necrotic cell debris was present, and irregularly arranged connective tissue was seen in the lamina propria, with pronounced inflammatory cell infiltration. The RIOM+M group showed signs of early reepithelialization, although some inflammatory cell infiltration persisted. In the RIOM+H group, reepithelialization was also initiated, but the restoration was incomplete, with residual inflammatory cell infiltration. Representative HE-stained tissue sections are shown in **Figure 5A**. The corresponding HE staining scores are presented in **Figure 5B**, **Table 3**. Compared to the RIOM group, the RIOM+L, RIOM+M, RIOM+H, and RIOM+K treatments resulted in significantly better HE staining scores (*P* < 0.05, *P* < 0.01, or *P* < 0.001).

**Table 3:** HE staining score for each group.

Group	Index value (M (p25, p75))	H value	p-value
Control	0.00 (0.00,0.00)###	34.10	p < 0.001
RIOM	3.20 (3.10,3.30)***		
RIOM+K	0.65 (0.50,0.80)###		
RIOM+L	2.60 (2.50,2.90)***		
RIOM+M	1.55 (1.50,1.80)**#		
RIOM+H	0.95 (0.90,1.10)##		

*P < 0.05, **P < 0.01, ***P < 0.001 vs. Control; #P < 0.05, ##P < 0.01, ###P < 0.001 vs. RIOM.

#### 3.3.3 HDZJF reduces the inflammatory factor levels in RIOM

**Figure 6:**
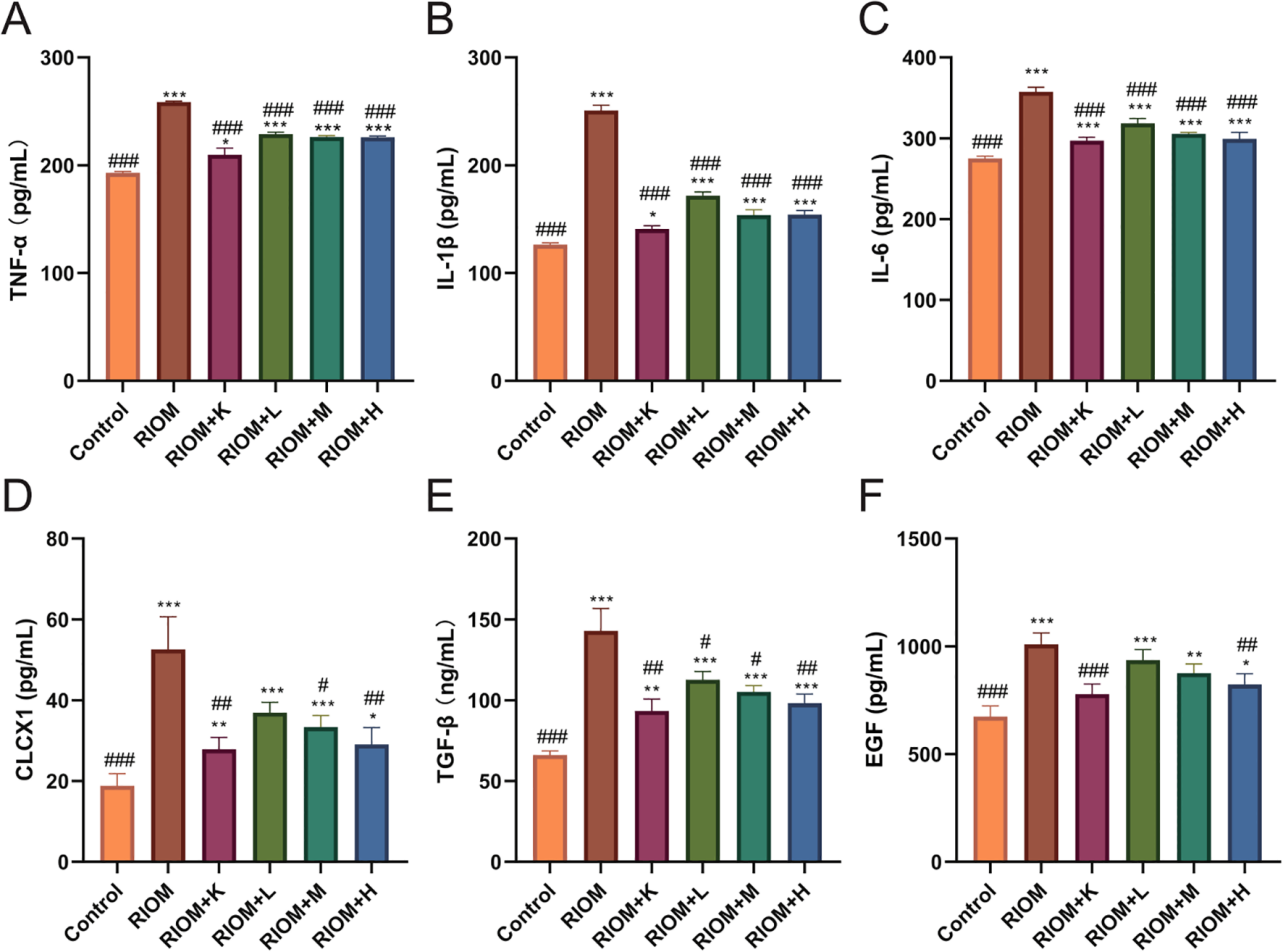
Effect of HDZJF on serum cytokine levels in rats: **(A)** TNF-α, **(B)** IL-1β, **(C)** IL-6, **(D)** CLCX1, **(E)** TGF-β, **(F)** EGF. n = 6 per group. The data are presented as mean ± SEM, with n = 6 per group. *P < 0.05, **P < 0.01, ***P < 0.001 vs. Control; #*P* < 0.05, ##*P* < 0.01, ###*P* < 0.001 vs. RIOM.

The serum levels of TNF-α, IL-1β, IL-6, CLCX1, TGF-β, and EGF were measured using the ELISA method. As shown in **Figure 6**, **Table 4**, compared to the Control group, the serum levels of TNF-α, IL-1β, IL-6, CLCX1, and TGF-β were significantly elevated in the other groups (*P* < 0.05, *P* < 0.01, or *P* < 0.001). Serum EGF levels in the RIOM+K group did not differ significantly from those in the Control group. Compared to the RIOM group, serum levels of TNF-α, IL-1β, and IL-6 were significantly reduced in the other groups (*P* < 0.001). The RIOM+K, RIOM+M, and RIOM+H groups significantly reduced serum CLCX1 levels, while the reduction in CLCX1 levels in the RIOM+L group was not statistically significant. Compared to the RIOM group, serum levels of TGF-β were significantly decreased in the other groups (*P* < 0.05, *P* < 0.01, or *P* < 0.001). Serum EGF levels were reduced in the RIOM+L and RIOM+M groups, but the differences were not statistically significant. In contrast, serum EGF levels in the RIOM+H group were significantly reduced (*P* < 0.01). In summary, HDZJF can effectively reduce the levels of inflammatory factors in serum within a certain concentration range.

**Table 4:** Serum cytokine levels for each group in rats.

Index	Group	Index value (mean ± SEM or M (p25, p75))	95%CI	F value (one-way ANOVA)	p-value	H value	p-value
TNF-α (pg/mL)	Control	193.00 ± 1.28###	(191.66,194.34)	1821.47	p < 0.001	–	–
	RIOM	258.53 ± 0.93***	(257.55,259.51)				
	RIOM+K	209.76 ± 6.01*###	(203.46,216.07)				
	RIOM+L	228.98 ± 1.67***###	(227.22,230.73)				
	RIOM+M	226.25 ± 1.15***###	(225.04,227.45)				
	RIOM+H	226.10 ± 0.99***###	(225.05,227.14)				
IL-1β (pg/mL)	Control	193.00 ± 1.28###	(191.66,194.34)	1821.47	p < 0.001	–	–
	RIOM	258.53 ± 0.93***	(257.55,259.51)				
	RIOM+K	209.76 ± 6.01*###	(203.46,216.07)				
	RIOM+L	228.98 ± 1.67***###	(227.22,230.73)				
	RIOM+M	226.25 ± 1.15***###	(225.04,227.45)				
	RIOM+H	226.10 ± 0.99***###	(225.05,227.14)				
IL-6 (pg/mL)	Control	275.06 ± 2.90###	(272.02,278.10)	180.78	p < 0.001	–	–
	RIOM	357.58 ± 5.49***	(351.82,363.34)				
	RIOM+K	297.09 ± 4.16***###	(292.72,301.46)				
	RIOM+L	318.45 ± 5.92***###	(312.24,324.66)				
	RIOM+M	305.49 ± 1.76***###	(303.65,307.34)				
	RIOM+H	299.49 ± 7.70***###	(291.41,307.57)				
CLCX1 (pg/mL)	Control	18.85 ± 2.97###	(15.73,21.97)	31.14	p < 0.001	–	–
	RIOM	52.58 ± 8.11***	(44.07,61.08)				
	RIOM+K	27.87 ± 2.93**##	(24.80,30.94)				
	RIOM+L	36.93 ± 2.56***	(34.24,39.62)				
	RIOM+M	33.32 ± 2.89***#	(30.28,36.35)				
	RIOM+H	29.07 ± 4.15*##	(24.71,33.42)				
TGF-β (ng/mL)	Control	66.15 ± 2.57###	(63.45,68.84)	137.31	p < 0.001	–	–
	RIOM	142.92 ± 13.88***	(128.36,157.49)				
	RIOM+K	93.35 ± 7.33**##	(85.66,101.05)				
	RIOM+L	112.68 ± 5.06***#	(107.37,117.99)				
	RIOM+M	105.27 ± 3.70***#	(101.39,109.16)				
	RIOM+H	98.19 ± 5.58***##	(92.33,104.04)				
EGF (pg/mL)	Control	661.79 (628.11,720.20)###	–	–	–	30.71	p < 0.001
	RIOM	1006.79 (973.48,1023.18)***	–				
	RIOM+K	753.78 (745.49,834.36)###	–				
	RIOM+L	933.91 (899.23,973.48)***	–				
	RIOM+M	865.90 (840.99,899.23)**	–				
	RIOM+H	827.90 (777.50,868.41)*##	–				

*P < 0.05, **P < 0.01, ***P < 0.001 vs. Control; #P < 0.05, ##P < 0.01, ###P < 0.001 vs. RIOM.

#### 3.3.4 HDZJF inhibits the activation of the EGFR/PI3K/AKT signaling pathway

**Figure 7:**
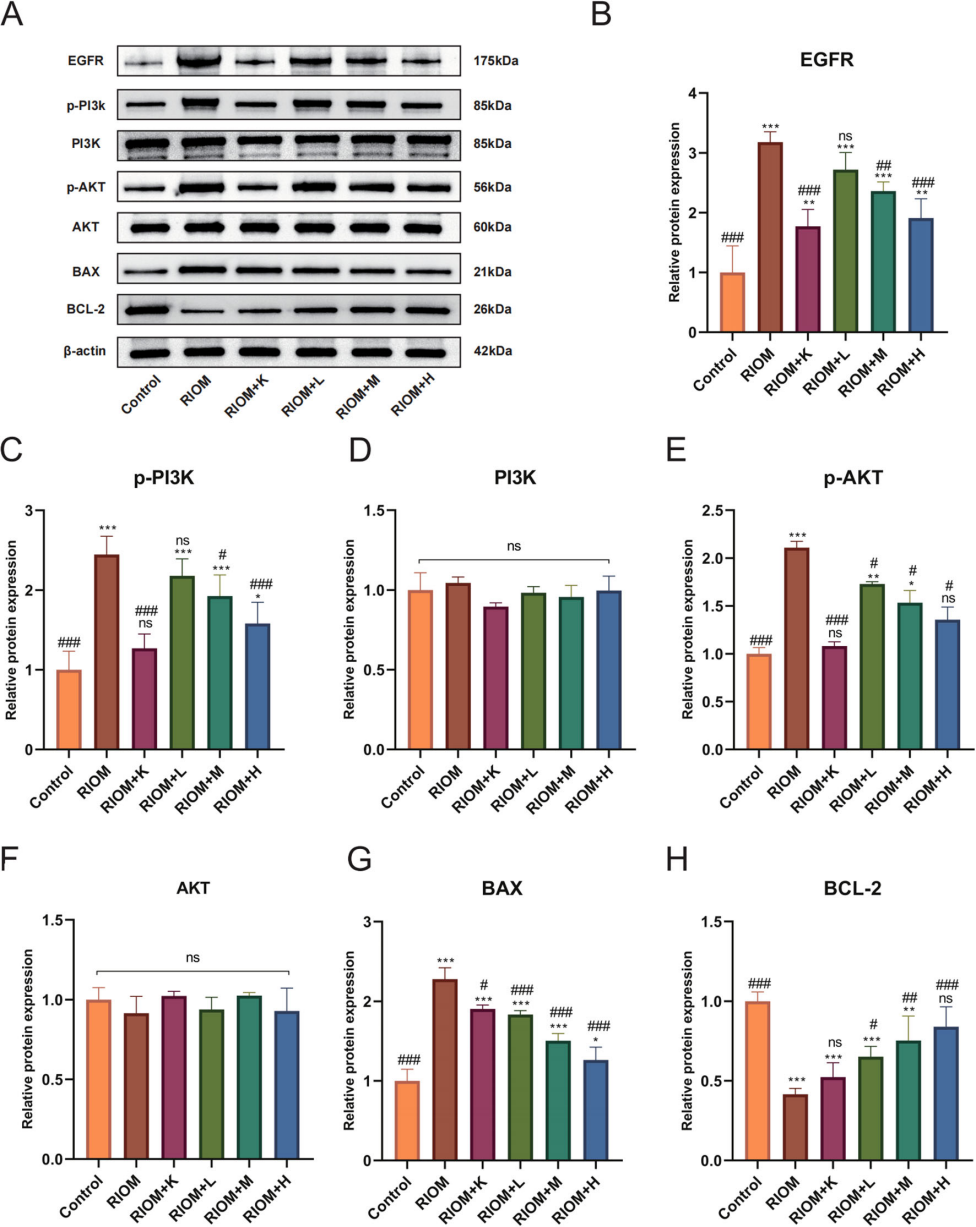
HDZJF inhibits the activation of the radiation-induced EGFR/PI3K/AKT signaling pathway. **(A)** Comparison of the expression of EGFR/PI3K/AKT signaling pathway proteins and apoptosis-related proteins among all groups. **(B-H)** Immunoblot analysis of tongue mucosal tissue was performed using specific antibodies against EGFR, p-PI3K, PI3K, p-AKT, AKT, BAX, BCL-2, and β-actin. The results were then normalized to β-actin. The data are presented as mean ± SEM, with n = 3 per group. *P < 0.05, **P < 0.01, ***P < 0.001 vs. Control; #P < 0.05, ##P < 0.01, ###P < 0.001 vs. RIOM; ns: not significant vs. Control or RIOM.

As shown in **Figure 7**, **Table 5**, compared to the control group, the expression of EGFR, p-PI3K, p-AKT, and BAX proteins in the tongue tissue of rats in the model group was markedly elevated (*P* < 0.001). In contrast, the expression of BCL-2 protein was significantly decreased (*P* < 0.001), showing an opposite trend to that of the other proteins. Compared to the RIOM group, the levels of EGFR, p-PI3K, p-AKT, and BAX proteins were markedly reduced, while BCL-2 protein expression was notably elevated in the tongue tissue of rats in the RIOM+L, RIOM+M, RIOM+H groups and RIOM+K (*P* < 0.05, *P* < 0.01, or *P* < 0.001). Furthermore, the higher the concentration of HDZJF, the more pronounced the changes.

**Table 5:** Expression of EGFR/PI3K/AKT signaling pathway proteins and apoptosis-related proteins for each group.

	Group	value (mean ± SEM or M (p25, p75))	95%CI	F value (one-way ANOVA)	p-value	H value	p-value
EGFR/β-ACTIN	Control	1.00 ± 0.45###	(-0.11,2.11)	20.77	p < 0.001	–	–
	RIOM	3.18 ± 0.17***	(2.75,3.61)				
	RIOM+K	1.77 ± 0.28**###	(1.07,2.47)				
	RIOM+L	2.72 ± 0.28***	(2.02,3.42)				
	RIOM+M	2.36 ± 0.15***##	(1.99,2.74)				
	RIOM+H	1.91 ± 0.32**###	(1.11,2.71)				
p-PI3K/β-ACTIN	Control	1.00 ± 0.23###	(0.42,1.58)	16.89	p < 0.001	–	–
	RIOM	2.45 ± 0.23***	(1.88,3.01)				
	RIOM+K	1.27 ± 0.18###	(0.82,1.72)				
	RIOM+L	2.18 ± 0.21***	(1.65,2.71)				
	RIOM+M	1.93 ± 0.27***#	(1.27,2.58)				
	RIOM+H	1.58 ± 0.27*###	(0.91,2.24)				
PI3K/β-ACTIN	Control	1.00 ± 0.11	(0.73,1.27)	1.59	0.24	–	–
	RIOM	1.04 ± 0.04	(0.95,1.14)				
	RIOM+K	0.90 ± 0.02	(0.84,0.95)				
	RIOM+L	0.98 ± 0.04	(0.89,1.08)				
	RIOM+M	0.96 ± 0.07	(0.78,1.13)				
	RIOM+H	1.00 ± 0.09	(0.77,1.22)				
p-AKT/β-ACTIN	Control	0.97 (0.96,102)###	–	–	–	16.06	0.007
	RIOM	2.12 (2.08,2.14)***	–				
	RIOM+K	1.10 (1.06,1.11)###	–				
	RIOM+L	1.73 (1.72,1.74)**#	–				
	RIOM+M	1.46 (1.46,1.57)*#	–				
	RIOM+H	1.28 (1.28,1.40)#	–				
AKT/β-ACTIN	Control	1.00 ± 0.07	(0.81,1.19)	1.01	0.45	–	–
	RIOM	1.02 ± 0.03	(0.95,1.09)				
	RIOM+K	0.94 ± 0.08	(0.75,1.13)				
	RIOM+L	1.03 ± 0.02	(0.98,1.07)				
	RIOM+M	0.93 ± 0.14	(0.57,1.28)				
	RIOM+H	0.92 ± 0.10	(0.66,1.17)				
BAX/β-ACTIN	Control	1.00 ± 0.15###	(0.64,1.36)	48.79	p < 0.001	–	–
	RIOM	2.28 ± 0.14***	(1.93,2.63)				
	RIOM+K	1.91 ± 0.05***#	(1.79,2.02)				
	RIOM+L	1.83 ± 0.05***###	(1.71,1.96)				
	RIOM+M	1.50 ± 0.09***###	(1.28,1.73)				
	RIOM+H	1.26 ± 0.16*###	(0.86,1.66)				
BCL-2/β-ACTIN	Control	1.00 ± 0.06###	(0.86,1.14)	14.57	p < 0.001	–	–
	RIOM	0.42 ± 0.04***	(0.32,0.51)				
	RIOM+K	0.52 ± 0.09***	(0.30,0.75)				
	RIOM+L	0.65 ± 0.06***#	(0.49,0.81)				
	RIOM+M	0.75 ± 0.15**##	(0.37,1.13)				
	RIOM+H	0.84 ± 0.12###	(0.53,1.15)				

*P < 0.05, **P < 0.01, ***P < 0.001 vs. Control; #P < 0.05, ##P < 0.01, ###P < 0.001 vs. RIOM.

## 4 Discussion

The pathogenesis of RIOM is considered a complex, multi-stage process. In the 1990s, oral medicine expert Stephen T. Sonis proposed a four-stage model of the pathophysiology of oral mucositis. Subsequent research refined this model into five stages, now widely accepted as Sonis’ five-stage pathological model: initiation, signaling, amplification, ulceration, and healing (43). During the initiation phase, radiation or chemotherapy causes DNA and non-DNA damage, leading to the production of reactive oxygen species, which disrupt biomolecules and trigger cell apoptosis. The signaling phase involves the activation of complex mechanisms induced by DNA and non-DNA damage, such as NF-κB activation, which results in gene upregulation and the release of pro-inflammatory cytokines. In the amplification phase, NF-κB is activated in various cell types, increasing pro-inflammatory cytokines and activating additional signaling pathways, ultimately leading to tissue damage. During the ulceration phase, bacterial colonization and sepsis risk occur, with Candida potentially exacerbating inflammation. The healing phase begins after the signals that induce persistent mucosal damage subside, allowing the mucosa to gradually heal. According to Sonis et al., the cascade effect mediated by cytokines secreted by macrophages and lymphocytes is a key factor in the onset and persistence of RIOM, with IL-6, IL-8, TNF-α, TGF-β, BAX, and BCL-2 playing significant roles in this process (44).

With the advancement of modern technology and in-depth research, significant advantages have been observed in areas such as productivity, reduced toxicity, multi-pathway targeting, multi-target effects, and microenvironment modulation (45). At the same time, TCM formulas and their active components have demonstrated considerable therapeutic potential in the treatment of various diseases, particularly in the fields of anti-inflammatory, antioxidant stress response, immune modulation, anti-tumor, cardiovascular protection, and neurological disorders, showing remarkable therapeutic effects and benefits (46–51).

Based on network pharmacology and molecular docking analyses, the therapeutic effects of HDZJF in treating RIOM appear to primarily result from its ability to improve the immune microenvironment, along with its anti-inflammatory, antioxidant, and anti-apoptotic properties. These actions collectively contribute to alleviating the underlying pathological processes associated with the condition. Building on this, we hypothesize that HDZJF may improve radiation-induced mucositis by modulating multiple key molecules, such as EGFR, PI3K, AKT, and downstream targets like NF-κB, BAX, and BCL-2. We further explored the mechanisms through which the EGFR/PI3K/AKT pathway may regulate RIOM.

EGFR, located on the cell membrane, is a member of the receptor tyrosine kinase (RTK) EGFR/ErbB subfamily. EGFR is activated by inflammatory factors such as IL-6, IL-1β, TNF-α, TGF-β, and EGF, leading to dimerization, which induces its own phosphorylation as well as the phosphorylation of the downstream PI3K, thereby activating the PI3K/AKT signaling pathway. A decrease in EGFR expression inhibits the activity of the PI3K/AKT signaling pathway, resulting in a series of downstream signaling events that regulate various biological processes such as cell survival, growth, proliferation, and apoptosis (52). A large body of literature has indicated that the EGFR/PI3K/AKT signaling pathway is involved in the regulation of oxidative stress, inflammatory responses, and autophagy, playing significant roles in balancing pro-inflammatory and anti-inflammatory factors, repairing the mucosal barrier, and modulating microbial balance (53–55).

We established a rat model of RIOM using an X-ray biological irradiation instrument, which provides a reliable and reproducible method for inducing ulceration in the oral mucosa. In our study, we observed that, compared to the RIOM group, the treatment group receiving HDZJF demonstrated a significant reduction in body weight loss. This result suggests that HDZJF might help improve the rats’ overall nutritional intake, promoting better food consumption and enhancing their general well-being. The reduction in body weight loss also indicates a potential improvement in the animals’ systemic health, which could be attributed to the anti-inflammatory and protective effects of the treatment. Macroscopic examination of the tongues further corroborated these findings. The treatment group showed a substantial decrease in the severity of ulceration compared to the RIOM group. The ulcers in the HDZJF-treated rats were smaller and less severe, indicating that HDZJF can effectively alleviate the symptoms of oral mucositis, likely by reducing inflammation and promoting mucosal healing. These observations suggest that HDZJF may have a protective role in mitigating the inflammatory damage caused by radiation, improving the recovery of the oral mucosa and enhancing the quality of life for affected individuals.

**Figure 8:**
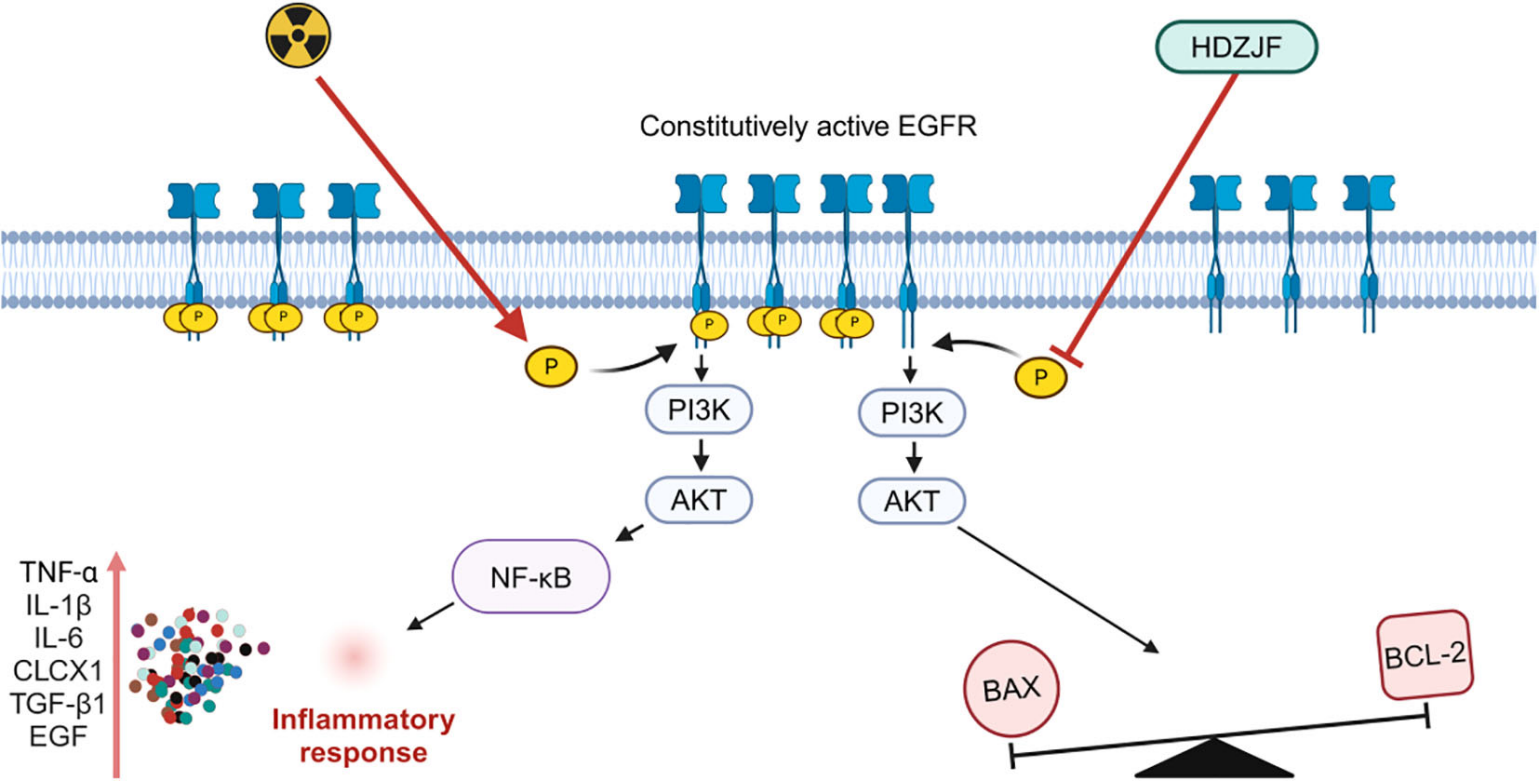
Mechanism of action of HDZJF in mitigating RIOM by targeting the EGFR/PI3K/AKT signaling pathway.

At the molecular level, we investigated the expression of key proteins involved in cell survival, apoptosis, and inflammatory signaling. We found that the expression of EGFR, p-PI3K, p-AKT, and BAX proteins was notably lower in the treatment group, while the anti-apoptotic protein BCL-2 showed significantly higher expression. By reducing the expression of pro-apoptotic proteins like BAX and increasing anti-apoptotic proteins like BCL-2, HDZJF likely helps to prevent cell death in the oral mucosa, promoting tissue regeneration and reducing ulcer severity. These findings indicate that HDZJF might protect against radiation-induced tissue damage by modulating the EGFR/PI3K/AKT signaling pathway, which is crucial for cell survival, proliferation, and resistance to apoptosis, as shown in **Figure 8**.

Compared to other RIOM treatment strategies, such as corticosteroids and local trauma care, the unique combination of these herbs in HDZJF offers a safer and more sustainable treatment option. It is worth noting that Dendrobium huoshanense, Ophiopogon japonicus, Rehmannia glutinosa, and Glycyrrhiza uralensis are all herbs that are commonly used in traditional Chinese medicine as both food and medicine. These herbs have been shown to possess anti-inflammatory, anti-tumor, antioxidant, and immune-regulatory properties (56–59). Specifically, Dendrobium huoshanense has been proven to regulate mucosal barrier function, while Glycyrrhiza uralensis has been confirmed to help prevent various oral diseases (60, 61). The herbal formulation in HDZJF may effectively address multiple aspects of RIOM, including pain relief, mucosal protection, and immune system regulation—factors crucial for cancer patients—making it a promising new therapeutic approach for the treatment of RIOM.

## 5 Limitations

Despite the valuable insights provided by this study, several limitations should be acknowledged. First, the experimental findings were derived from preclinical models, and clinical observations from human trials are currently lacking. This restricts direct extrapolation of the results to clinical scenarios. Second, the public databases utilized to identify bioactive components of HDZJF and RIOM-related target genes may not be exhaustive, potentially missing undocumented compounds and pathway interactions. Furthermore, additional research is required to confirm other potential targets and signaling pathways through which HDZJF may influence RIOM. Nevertheless, this study offers initial perspectives on the mechanisms of HDZJF in treating RIOM and serves as a basis for future exploration of new therapeutic approaches for RIOM.

## 6 Conclusion

Our findings suggest that HDZJF effectively prevents RIOM by inhibiting the EGFR/PI3K/AKT signaling pathway. This indicates that the EGFR/PI3K/AKT pathway holds significant potential as a therapeutic target for the prevention and treatment of RIOM.

## Data Availability

The datasets presented in this study can be found in online repositories. The names of the repository/repositories and accession number(s) can be found in the article/**Supplementary Material**.
