# Supplementary material for: Huoshan Dendrobium Zengye Jiedu Formula mitigates radiation-induced oral mucositis and improves oral immune microenvironment by targeting the EGFR/PI3K/AKT pathway: evidence from network pharmacology, molecular docking, and experimental validation
Source: Front Immunol. 2025 Mar 10;16:1559400. doi: 10.3389/fimmu.2025.1559400 (PMC11931053; doi:10.3389/fimmu.2025.1559400)
Supplement: Supplementary file 1 [file DataSheet1.docx]

Supplementary Material

**1.Supplementary Figure**

**
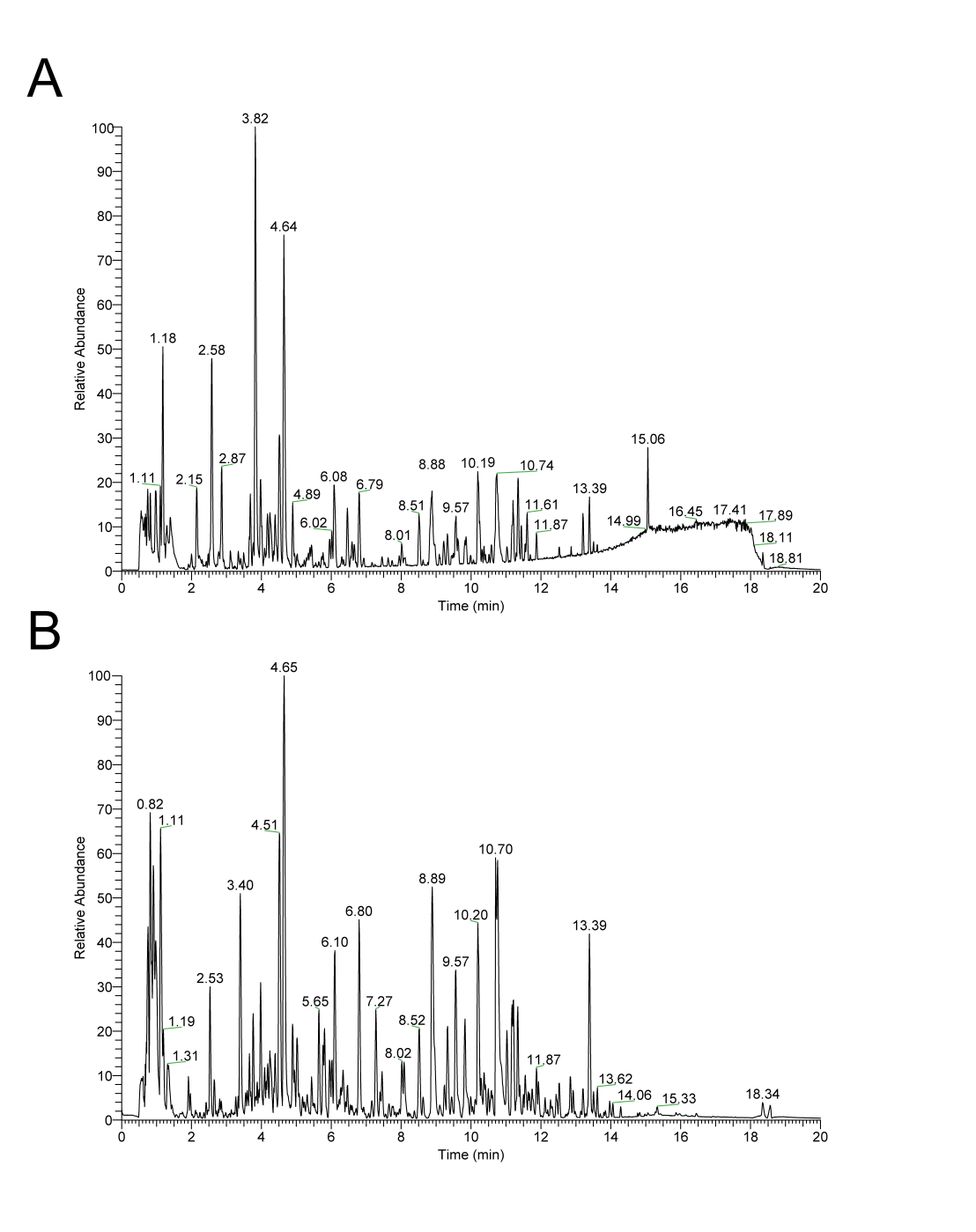
**

**Supplementary Figure 1. Total ion current diagram of HDZJF.** (A) The above figure means total ion flow diagram of the positive ion pattern. (B) The below figure means total ion flow diagram of the negative ion mode.

Weigh 100 mg of sample powder into a 1.5 mL centrifuge tube. Add 1 mL of 70% methanol, vortex for 30 s, then ultrasonicate for 90 min. Centrifuge at 4°C for 10 min at 16,000 g. Transfer the supernatant to a 96-well protein filter plate and filter under nitrogen pressure. Move the filtered solution to a 2 mL EP tube and dry under vacuum. Resuspend in 300 μL of 40% methanol, vortex for 30 s, and centrifuge again at 4°C for 10 min at 16,000 g. Use the supernatant for analysis. Chromatographic separation is performed using a Vanquish UHPLC system with an ACQUITY UPLC HSS T3 column (2.1 mm×100 mm, 1.8 µm). The column temperature is maintained at 35°C with a flow rate of 0.3 mL/min. The mobile phase consists of A: 0.1% formic acid in water and B: 0.1% formic acid in acetonitrile. Gradient elution is as follows:

| Time (minutes) | Mobile Phase A (%) | Mobile Phase B (%) |
| --- | --- | --- |
| Initial | 95 | 5 |
| 17.0 | 2 | 98 |
| 17.2 | 95 | 5 |
| 20.0 | 95 | 5 |

Mass spectrometry is conducted using a Q-Exactive HFX mass spectrometer coupled with the UHPLC system. The instrument operates in both positive and negative ESI modes, with spray voltages of 3800 V (ESI+) and 3500 V (ESI-). Sheath gas pressure is set at 45 arbitrary units, auxiliary gas pressure at 20 arbitrary units, ion transfer tube temperature at 320°C, and atomization temperature at 350°C. The detection mode is Full-MS/dd-MS2, with resolutions of 60,000 and 15,000 for primary and secondary stages, respectively. The top 10 MS1 ions are selected for MS/MS spectra, with collision energies normalized to 20, 40, and 60. The scan range for primary m/z is 90~1300. For analysis, 2 μL of HDZJF solution is injected into the LC-MS system, with the sample being analyzed five times to ensure reproducibility.

**2. Supplemetary Table**

**In the chromatograms of both positive and negative ion modes, peaks with higher abundance were confirmed for their shapes and inspected through secondary spectra. A total of 38 high-abundance peaks were identified, with 22 peaks in positive ion mode and 19 peaks in negative ion mode.The table is divided into positive and negative ion modes to display the different compounds detected under each mode.**

**Table 1 Positive Ion Mode:**

| No | m/z | RT/min | ppm | Formula | compound name | adduct | score | SuperClass |
| --- | --- | --- | --- | --- | --- | --- | --- | --- |
| 1 | 166.0864 | 2.15 | 1.5 | C13H17N3O4 | Gly-Gly-Phe | [M+H-C4H6N2O2]+ | 0.9991 | Small peptides |
| 2 | 262.1917 | 2.58 | 6.2 | C14H28ClNO | 2-Chloro-N-dodecylacetamide | [M+H]+ | 0.997 | Fatty amides |
| 3 | 188.0711 | 2.86 | 0.9 | C11H12N2O2 | Tryptophan | [M+H-NH3]+ | 0.9712 | Small peptides |
| 4 | 595.1664 | 3.66 | 3.2 | C27H30O15 | Vicenin-2 | [M+H]+ | 0.9844 | Flavonoids |
| 5 | 310.2014 | 3.83 | 0.3 | C17H27NO4 | Nadolol | [M+H]+ | 0.9784 | NA |
| 6 | 565.1559 | 3.98 | 2.3 | C26H28O14 | 4H-1-Benzopyran-4-one, 6-arabinopyranosyl-8-.beta.-D-glucopyranosyl-5,7-dihydroxy-2-(4-hydroxyphenyl)- | [M+H]+ | 0.9562 | Flavonoids |
| 7 | 294.2067 | 4.16 | 0.8 | C17H27NO3 | Pramacort | [M+H]+ | 0.992 | NA |
| 8 | 181.0862 | 4.25 | 1.7 | C12H19NO3 | Escaline | [M+H-C2H7N]+ | 0.7028 | Tyrosine alkaloids |
| 9 | 535.1454 | 4.39 | 1.5 | C25H26O13 | 4H-1-Benzopyran-4-one, 5,7-dihydroxy-2-(4-hydroxyphenyl)-6,8-dipentopyranosyl- | [M+H]+ | 0.9664 | Flavonoids |
| 10 | 257.081 | 4.51 | 0.9 | C21H22O9 | Neoliquiritin | [M+H-C6H10O5]+ | 0.9939 | Flavonoids |
| 11 | 419.1342 | 4.64 | 0 | C21H22O9 | Liquiritin | [M+H]+ | 0.9953 | Flavonoids |
| 12 | 401.1601 | 4.9 | 1.3 | C22H26O8 | Syringaresinol | [M+H-H2O]+ | 0.8751 | Lignans |
| 13 | 431.1343 | 6.09 | 1.7 | C22H22O9 | Ononin | [M+H]+ | 0.9996 | Isoflavonoids |
| 14 | 314.1392 | 6.46 | 0.6 | C18H19NO4 | Moupinamide | [M+H]+ | 0.9422 | Phenylpropanoids (C6-C3) |
| 15 | 257.0813 | 6.8 | 2.3 | C27H32O14 | Glucoliquiritin | [M+H-C12H20O10]+ | 0.9935 | Flavonoids |
| 16 | 511.3424 | 8.9 | 6.4 | C30H48O5 | Alisol F | [M+Na]+ | 0.9759 | Triterpenoids |
| 17 | 839.4064 | 10.2 | 0.1 | C42H62O17 | Licoricesaponin g2 | [M+H]+ | 0.9372 | Triterpenoids |
| 18 | 453.3368 | 10.74 | 1.4 | C30H46O4 | 18.beta.-Glycyrrhetinic acid | [M+H-H2O]+ | 0.9942 | Triterpenoids |
| 19 | 273.1125 | 11.21 | 1.2 | C16H18O5 | (.+/-.)-Murracarpin | [M+H-H2O]+ | 0.9725 | Coumarins |
| 20 | 286.1441 | 11.34 | 1.4 | C17H19NO3 | Piperine | [M+H]+ | 0.9853 | Lysine alkaloids |
| 21 | 520.3403 | 13.2 | 0.4 | C26H50NO7P | LPC 18:2 | [M+H]+ | 0.9604 | Glycerophospholipids |
| 22 | 542.3224 | 13.38 | 1.7 | C26H50NO7P | .beta.-Linoleoyl-.alpha.-glycerophosphorylcholine | [M+Na]+ | 0.9204 | Glycerophospholipids |

**Table 2 Negative Ion Mode:**

| No | m/z | RT/min | ppm | Formula | compound name | adduct | score | SuperClass |
| --- | --- | --- | --- | --- | --- | --- | --- | --- |
| 1 | 393.1418 | 2.53 | 3.5 | C15H24O9 | Ajugol | [M+HCO2]- | 0.8881 | Monoterpenoids |
| 2 | 165.0558 | 3.4 | 4.4 | C9H10O3 | Benzenepropanoic acid, 4-hydroxy- | [M-H]- | 0.9978 | NA |
| 3 | 593.1539 | 3.66 | 5.8 | C27H30O15 | Vicenin-2 | [M-H]- | 0.9863 | Flavonoids |
| 4 | 401.147 | 3.77 | 6.0 | C18H26O10 | Benzyl alcohol + hex-pen | [M-H]- | 0.8928 | NA |
| 5 | 563.1431 | 3.98 | 4.7 | C26H28O14 | Schaftoside | [M-H]- | 0.9937 | Flavonoids |
| 6 | 549.1637 | 4.51 | 4 | C26H30O13 | 4-(7-Hydroxy-4-oxo-3,4-dihydro-2H-chromen-2-yl)phenyl 2-O-(3,4-dihydroxy-4-(hydroxymethyl)tetrahydrofuran-2-yl)hexopyranoside | [M-H]- | 0.9741 | Flavonoids |
| 7 | 417.1206 | 4.65 | 3.7 | C21H22O9 | Liquiritin | [M-H]- | 0.9816 | Flavonoids |
| 8 | 579.2111 | 4.9 | 4.9 | C28H36O13 | (-)-Syringaresinol-4-O-.beta.-D-glucopyranoside | [M-H]- | 0.9528 | Lignans |
| 9 | 459.1529 | 5.81 | 3.1 | C20H28O12 | Paeonolide | [M-H]- | 0.9248 | Phloroglucinols |
| 10 | 417.1208 | 6.1 | 5.2 | C21H22O9 | Isoliquiritin | [M-H]- | 0.9563 | Flavonoids |
| 11 | 255.0673 | 6.8 | 4.7 | C15H12O4 | Isoliquiritigen | [M-H]- | 0.9692 | Flavonoids |
| 12 | 329.2348 | 9.88 | 4.6 | C18H34O5 | 9-Octadecenoic acid, 5,8,11-trihydroxy- | [M-H]- | 0.9902 | Octadecanoids |
| 13 | 837.3943 | 10.21 | 3.0 | C42H62O17 | Licoricesaponin g2 | [M-H]- | 0.9907 | Triterpenoids |
| 14 | 821.3993 | 10.75 | 3.3 | C42H62O16 | Licoricesaponin h2 | [M-H]- | 0.991 | Triterpenoids |
| 15 | 807.4204 | 11.03 | 3.0 | C42H64O15 | Licoricesaponin B2 | [M-H]- | 0.9761 | Triterpenoids |
| 16 | 821.3998 | 11.17 | 4.0 | C42H62O16 | (2S,3S,4S,5R,6R)-6-[(2R,3R,4S,5S,6S)-2-[[(3S,6aR,6bS,8aS,12aR,14bS)-11-carboxy-4,4,6a,6b,8a,11,14b-heptamethyl-14-oxo-2,3,4a,5,6,7,8,9,10,12,12a,14a-dodecahydro-1H-picen-3-yl]oxy]-6-carboxy-4,5-dihydroxyoxan-3-yl]oxy-3,4,5-trihydroxyoxane-2-carboxylic acid | [M-H]- | 0.9811 | Triterpenoids |
| 17 | 271.0987 | 11.21 | 3.5 | C22H28O10 | 5-O-Methylvisammioside | [M-H-C6H12O6]- | 0.7412 | Chromanes |
| 18 | 367.12 | 11.34 | 3.4 | C21H20O6 | Glycycoumarin | [M-H]- | 0.9973 | Coumarins |
| 19 | 295.2288 | 13.39 | 2.9 | C18H32O3 | 12(13)-EpOME | [M-H]- | 0.9963 | Fatty Acids and Conjugates |

**Note: NO: Serial number; m/z: Parent ion mass-to-charge ratio; RT/min: Retention time in minutes; ppm: Mass deviation at the first stage; Score: Matching score of the second stage mass spectrum; SuperClass: Compound classification; NA: Not available.**
