# Supplementary material for: Huoshan Dendrobium Zengye Jiedu Formula mitigates radiation-induced oral mucositis and improves oral immune microenvironment by targeting the EGFR/PI3K/AKT pathway: evidence from network pharmacology, molecular docking, and experimental validation
Source: Front Immunol. 2025 Mar 10;16:1559400. doi: 10.3389/fimmu.2025.1559400 (PMC11931053; doi:10.3389/fimmu.2025.1559400)
Supplement: Supplementary file 2 [file Table1.docx]

Supplementary Table 1. The results of normality and homogeneity of variance tests for body weights.

| Group | Weight(g) (mean ± SEM ) | W value | p-value | F value  (Levene’s test) | p-value | χ^2^  (sphericity test) | p-value |
| --- | --- | --- | --- | --- | --- | --- | --- |
| Control | 327.83±14.72### | 0.97 | 0.49 | 19.40 | p < 0.001 | 788.60 | p < 0.001 |
| RIOM | 267.15±31.59*** | 0.97 | 0.51 |  |  |  |  |
| RIOM+K | 284.53±19.38***# | 0.97 | 0.42 |  |  |  |  |
| RIOM+L | 282.65±19.21*** | 0.98 | 0.62 |  |  |  |  |
| RIOM+M | 284.33±20.02***# | 0.97 | 0.48 |  |  |  |  |
| RIOM+H | 295.26±15.51***### | 0.96 | 0.28 |  |  |  |  |

*P < 0.05, **P < 0.01, ***P < 0.001 vs. Control; #P < 0.05, ##P < 0.01, ###P < 0.001 vs. RIOM.
