# Supplementary material for: Huoshan Dendrobium Zengye Jiedu Formula mitigates radiation-induced oral mucositis and improves oral immune microenvironment by targeting the EGFR/PI3K/AKT pathway: evidence from network pharmacology, molecular docking, and experimental validation
Source: Front Immunol. 2025 Mar 10;16:1559400. doi: 10.3389/fimmu.2025.1559400 (PMC11931053; doi:10.3389/fimmu.2025.1559400)
Supplement: Supplementary file 3 [file Table2.docx]

Supplementary Table 2. The results of normality and homogeneity of variance tests for Oral Mucositis Scores on Days 9-14 in each group.

| Group | RIOM score (mean ± SEM ) | W value | p-value | F value  (Levene’s test) | p-value | χ^2^  (sphericity test) | p-value |
| --- | --- | --- | --- | --- | --- | --- | --- |
| Control | 0.00±0.00### | - | - | 24.76 | p < 0.001 | 37.89 | p < 0.001 |
| RIOM | 2.69±0.25*** | 0.92 | 0.01 |  |  |  |  |
| RIOM+K | 2.27±0.47***### | 0.93 | 0.03 |  |  |  |  |
| RIOM+L | 2.51±0.28***### | 0.94 | 0.05 |  |  |  |  |
| RIOM+M | 2.45±0.36***### | 0.91 | 0.01 |  |  |  |  |
| RIOM+H | 2.39±0.39***### | 0.95 | 0.10 |  |  |  |  |

*P < 0.05, **P < 0.01, ***P < 0.001 vs. Control; #P < 0.05, ##P < 0.01, ###P < 0.001 vs. RIOM.
