# Supplementary material for: Huoshan Dendrobium Zengye Jiedu Formula mitigates radiation-induced oral mucositis and improves oral immune microenvironment by targeting the EGFR/PI3K/AKT pathway: evidence from network pharmacology, molecular docking, and experimental validation
Source: Front Immunol. 2025 Mar 10;16:1559400. doi: 10.3389/fimmu.2025.1559400 (PMC11931053; doi:10.3389/fimmu.2025.1559400)
Supplement: Supplementary file 4 [file Table3.docx]

Supplementary Table 3. The results of normality and homogeneity of variance tests for HE staining scores in each group.

| Group | Index value (M (p25, p75)) | W value | p-value | F value  (Levene’s test) | p-value |
| --- | --- | --- | --- | --- | --- |
| Control | 0.00(0.00,0.00)### | - | - | 3.10 | 0.02 |
| RIOM | 3.20(3.10,3.30)*** | 0.93 | 0.57 |  |  |
| RIOM+K | 0.65(0.50,0.80)### | 0.86 | 0.19 |  |  |
| RIOM+L | 2.60(2.50,2.90)*** | 0.91 | 0.42 |  |  |
| RIOM+M | 1.55(1.50,1.80)**# | 0.96 | 0.79 |  |  |
| RIOM+H | 0.95(0.90,1.10)## | 0.96 | 0.80 |  |  |

*P < 0.05, **P < 0.01, ***P < 0.001 vs. Control; #P < 0.05, ##P < 0.01, ###P < 0.001 vs. RIOM.
