# Supplementary material for: Huoshan Dendrobium Zengye Jiedu Formula mitigates radiation-induced oral mucositis and improves oral immune microenvironment by targeting the EGFR/PI3K/AKT pathway: evidence from network pharmacology, molecular docking, and experimental validation
Source: Front Immunol. 2025 Mar 10;16:1559400. doi: 10.3389/fimmu.2025.1559400 (PMC11931053; doi:10.3389/fimmu.2025.1559400)
Supplement: Supplementary file 5 [file Table4.docx]

Supplementary Table 4. The results of normality and homogeneity of variance tests for serum cytokine levels in each group of rats.

| Index | Group | Index value  (mean ± SEM or M (p25, p75)) | W value | p-value | F value  (Levene’s test) | p-value |
| --- | --- | --- | --- | --- | --- | --- |
| TNF-α (pg/mL) | Control | 193.00±1.28### | 0.92 | 0.49 | 8.71 | p < 0.001 |
|  | RIOM | 258.53±0.93*** | 0.93 | 0.57 |  |  |
|  | RIOM+K | 209.76±6.01*### | 0.91 | 0.44 |  |  |
|  | RIOM+L | 228.98±1.67***### | 0.86 | 0.20 |  |  |
|  | RIOM+M | 226.25±1.15***### | 0.97 | 0.91 |  |  |
|  | RIOM+H | 226.10±0.99***### | 0.97 | 0.88 |  |  |
| IL-1β (pg/mL) | Control | 193.00±1.28### | 0.92 | 0.49 | 8.71 | p < 0.001 |
|  | RIOM | 258.53±0.93*** | 0.93 | 0.57 |  |  |
|  | RIOM+K | 209.76±6.01*### | 0.91 | 0.44 |  |  |
|  | RIOM+L | 228.98±1.67***### | 0.86 | 0.20 |  |  |
|  | RIOM+M | 226.25±1.15***### | 0.97 | 0.91 |  |  |
|  | RIOM+H | 226.10±0.99***### | 0.97 | 0.88 |  |  |
| IL-6 (pg/mL) | Control | 275.06±2.90### | 0.94 | 0.63 | 2.33 | 0.07 |
|  | RIOM | 357.58±5.49*** | 0.82 | 0.09 |  |  |
|  | RIOM+K | 297.09±4.16***### | 0.87 | 0.22 |  |  |
|  | RIOM+L | 318.45±5.92***### | 0.92 | 0.52 |  |  |
|  | RIOM+M | 305.49±1.76***### | 0.86 | 0.20 |  |  |
|  | RIOM+H | 299.49±7.70***### | 0.94 | 0.64 |  |  |
| CLCX1 (pg/mL) | Control | 18.85±2.97### | 0.91 | 0.46 | 2.79 | 0.04 |
|  | RIOM | 52.58±8.11*** | 0.97 | 0.88 |  |  |
|  | RIOM+K | 27.87±2.93**## | 0.91 | 0.45 |  |  |
|  | RIOM+L | 36.93±2.56*** | 0.86 | 0.18 |  |  |
|  | RIOM+M | 33.32±2.89***# | 0.88 | 0.26 |  |  |
|  | RIOM+H | 29.07±4.15*## | 0.84 | 0.13 |  |  |
| TGF-β (ng/mL) | Control | 66.15±2.57### | 0.88 | 0.28 | 2.67 | 0.04 |
|  | RIOM | 142.92±13.88*** | 0.98 | 0.93 |  |  |
|  | RIOM+K | 93.35±7.33**## | 0.94 | 0.66 |  |  |
|  | RIOM+L | 112.68±5.06***# | 0.89 | 0.31 |  |  |
|  | RIOM+M | 105.27±3.70***# | 0.97 | 0.87 |  |  |
|  | RIOM+H | 98.19±5.58***## | 0.86 | 0.18 |  |  |
| EGF (pg/mL) | Control | 661.79(628.11,720.20)### | 0.90 | 0.39 | 0.14 | 0.98 |
|  | RIOM | 1006.79(973.48,1023.18)*** | 0.94 | 0.67 |  |  |
|  | RIOM+K | 753.78(745.49,834.36)### | 0.74 | 0.02 |  |  |
|  | RIOM+L | 933.91(899.23,973.48)*** | 0.98 | 0.95 |  |  |
|  | RIOM+M | 865.90(840.99,899.23)** | 0.91 | 0.47 |  |  |
|  | RIOM+H | 827.90(777.50,868.41)*## | 0.87 | 0.23 |  |  |

*P < 0.05, **P < 0.01, ***P < 0.001 vs. Control; #P < 0.05, ##P < 0.01, ###P < 0.001 vs. RIOM.
