# Supplementary material for: Huoshan Dendrobium Zengye Jiedu Formula mitigates radiation-induced oral mucositis and improves oral immune microenvironment by targeting the EGFR/PI3K/AKT pathway: evidence from network pharmacology, molecular docking, and experimental validation
Source: Front Immunol. 2025 Mar 10;16:1559400. doi: 10.3389/fimmu.2025.1559400 (PMC11931053; doi:10.3389/fimmu.2025.1559400)
Supplement: Supplementary file 6 [file Table5.docx]

Supplementary Table 5. The results of normality and homogeneity of variance tests for expression levels of EGFR/PI3K/AKT signaling pathway proteins and apoptosis-related proteins in each group.

|  | Group | value  (mean ± SEM or M (p25, p75)) | W value | p-value | F value  (Levene’s test) | p-value |
| --- | --- | --- | --- | --- | --- | --- |
| EGFR/β-ACTIN | Control | 1.00±0.45### | 0.99 | 0.80 | 0.97 | 0.47 |
|  | RIOM | 3.18±0.17*** | 0.99 | 0.82 |  |  |
|  | RIOM+K | 1.77±0.28**### | 0.94 | 0.54 |  |  |
|  | RIOM+L | 2.72±0.28*** | 0.98 | 0.74 |  |  |
|  | RIOM+M | 2.36±0.15***## | 0.97 | 0.68 |  |  |
|  | RIOM+H | 1.91±0.32**### | 0.86 | 0.28 |  |  |
| p-PI3K/β-ACTIN | Control | 1.00±0.23### | 0.81 | 0.14 | 0.14 | 0.98 |
|  | RIOM | 2.45±0.23*** | 1.00 | 0.89 |  |  |
|  | RIOM+K | 1.27±0.18### | 0.93 | 0.51 |  |  |
|  | RIOM+L | 2.18±0.21*** | 0.79 | 0.10 |  |  |
|  | RIOM+M | 1.93±0.27***# | 0.99 | 0.85 |  |  |
|  | RIOM+H | 1.58±0.27*### | 0.96 | 0.62 |  |  |
| PI3K/β-ACTIN | Control | 1.00±0.11 | 1.00 | 0.98 | 0.98 | 0.47 |
|  | RIOM | 1.04±0.04 | 0.84 | 0.23 |  |  |
|  | RIOM+K | 0.90±0.02 | 0.88 | 0.33 |  |  |
|  | RIOM+L | 0.98±0.04 | 0.99 | 0.86 |  |  |
|  | RIOM+M | 0.96±0.07 | 1.00 | 0.94 |  |  |
|  | RIOM+H | 1.00±0.09 | 1.00 | 0.96 |  |  |
| p-AKT/β-ACTIN | Control | 0.97(0.96,102)### | 0.79 | 0.08 | 0.31 | 0.90 |
|  | RIOM | 2.12(2.08,2.14)*** | 0.98 | 0.71 |  |  |
|  | RIOM+K | 1.10(1.06,1.11)### | 0.87 | 0.29 |  |  |
|  | RIOM+L | 1.73(1.72,1.74)**# | 0.98 | 0.75 |  |  |
|  | RIOM+M | 1.46(1.46,1.57)*# | 0.77 | 0.04 |  |  |
|  | RIOM+H | 1.28(1.28,1.40)# | 0.79 | 0.10 |  |  |
| AKT/β-ACTIN | Control | 1.00±0.07 | 0.90 | 0.40 | 2.11 | 0.14 |
|  | RIOM | 1.02±0.03 | 0.86 | 0.28 |  |  |
|  | RIOM+K | 0.94±0.08 | 0.79 | 0.10 |  |  |
|  | RIOM+L | 1.03±0.02 | 0.96 | 0.62 |  |  |
|  | RIOM+M | 0.93±0.14 | 0.96 | 0.61 |  |  |
|  | RIOM+H | 0.92±0.10 | 1.00 | 0.99 |  |  |
| BAX/β-ACTIN | Control | 1.00±0.15### | 1.00 | 0.88 | 1.61 | 0.23 |
|  | RIOM | 2.28±0.14*** | 0.81 | 0.14 |  |  |
|  | RIOM+K | 1.91±0.05***# | 0.98 | 0.74 |  |  |
|  | RIOM+L | 1.83±0.05***### | 0.85 | 0.24 |  |  |
|  | RIOM+M | 1.50±0.09***### | 0.86 | 0.28 |  |  |
|  | RIOM+H | 1.26±0.16*### | 0.95 | 0.55 |  |  |
| BCL-2/β-ACTIN | Control | 1.00±0.06### | 0.97 | 0.69 | 1.21 | 0.36 |
|  | RIOM | 0.42±0.04*** | 0.98 | 0.70 |  |  |
|  | RIOM+K | 0.52±0.09*** | 0.79 | 0.08 |  |  |
|  | RIOM+L | 0.65±0.06***# | 1.00 | 0.95 |  |  |
|  | RIOM+M | 0.75±0.15**## | 0.99 | 0.83 |  |  |
|  | RIOM+H | 0.84±0.12### | 0.99 | 0.84 |  |  |

*P < 0.05, **P < 0.01, ***P < 0.001 vs. Control; #P < 0.05, ##P < 0.01, ###P < 0.001 vs. RIOM.
